# Broadening Humor: Comic Styles Differentially Tap into Temperament, Character, and Ability

**DOI:** 10.3389/fpsyg.2018.00006

**Published:** 2018-01-18

**Authors:** Willibald Ruch, Sonja Heintz, Tracey Platt, Lisa Wagner, René T. Proyer

**Affiliations:** ^1^Personality and Assessment, Department of Psychology, University of Zurich, Zurich, Switzerland; ^2^Institute of Psychology, University of Wolverhampton, Wolverhampton, United Kingdom; ^3^Department of Psychology, Martin-Luther University Halle-Wittenberg, Halle, Germany

**Keywords:** humor, ridicule, fun, satire, wit, irony, personality, character

## Abstract

The present study introduces eight *comic styles* (i.e., fun, humor, nonsense, wit, irony, satire, sarcasm, and cynicism) and examines the validity of a set of 48 marker items for their assessment, the Comic Style Markers (CSM). These styles were originally developed to describe literary work and are used here to describe individual differences. Study 1 examines whether the eight styles can be distinguished empirically, in self- and other-reports, and in two languages. In different samples of altogether more than 1500 adult participants, the CSM was developed and evaluated with respect to internal consistency, homogeneity, test–retest reliability, factorial validity, and construct and criterion validity. Internal consistency was sufficiently high, and the median test-retest reliability over a period of 1–2 weeks was 0.86 (*N* = 148). Exploratory and confirmatory factor analyses showed that the eight styles could be distinguished in both English- (*N* = 303) and German-speaking samples (*N* = 1018 and 368). Comparing self- and other-reports (*N* = 210) supported both convergent and discriminant validity. The intercorrelations among the eight scales ranged from close to zero (between humor and sarcasm/cynicism) to large and positive (between sarcasm and cynicism). Consequently, second-order factor analyses revealed either two bipolar factors (based on ipsative data) or three unipolar factors (based on normative data). Study 2 related the CSM to instruments measuring personality (*N* = 999), intelligence (*N* = 214), and character strengths (*N* = 252), showing that (a) wit was the only style correlated with (verbal) intelligence, (b) fun was related to indicators of vitality and extraversion, (c) humor was related to character strengths of the heart, and (d) comic styles related to mock/ridicule (i.e., sarcasm, cynicism, but also irony) correlated negatively with character strengths of the virtues temperance, transcendence, and humanity. By contrast, satire had a moral goodness that was lacking in sarcasm and cynicism. Most importantly, the two studies revealed that humor might be related to a variety of character strengths depending on the comic style utilized, and that more styles may be distinguished than has been done in the past. The CSM is recommended for future explorations and refinements of comic styles.

## Introduction

Humor research has to accommodate that people habitually differ in terms of humor behaviors both quantitatively (i.e., some show more humor production, appreciation, etc., than others) and qualitatively (i.e., people indulge in different forms in humor). The quantitative part was taken care of by regarding humor as a continuum ranging from low to high, rather than solely distinguishing between having it and not having it. The qualitative part acknowledges that humor might have a different tone (*comic tonality*), *flavor* (just as people have different tastes), form, type, distinctive quality, or *style*. These qualitative differences may reflect mood (e.g., cheerful or bitter), degree of refinement (e.g., from low comedy often based on physical incongruity to high comedy of manners with more emphasis on language), structure (i.e., genre) or modality (for an overview, see Ruch, 2008).

So far, these differences have not been studied. For a systematic classification of styles, we not only need to consider the different qualities (i.e., the horizontal level) but also different levels of abstraction (i.e., the vertical level). Humor styles might be very specific, representing a narrow scope of behaviors (e.g., teasing, bantering), or they might be more general, covering a broader range of different behaviors (e.g., socially warm humor). The lower in the level of abstraction, the closer one is to a form of behavior that could actually be shown in a specific situation or that could be trained or modified. Conversely, this is less likely the higher up the hierarchy a style is located, as more general styles are abstractions representing dispositions to behaviors. Thus, the more a style is a composite of many behaviors, the less easily it can be shown, trained, or modified as a block (in its entirety). Lower-level approaches to styles are more informative but also more redundant, while more abstract approaches are more parsimonious, but at the expense of detailed descriptions. Hence, a comprehensive approach needs to consider different levels of aggregations.

The two approaches to humor styles introduced in the past two decades can be located at an intermediate level. The Humorous Behavior Q-sort Deck (HBQD; Craik et al., 1996) distinguishes 10 styles of everyday humorous conduct that were allocated to five bipolar dimensions; namely, socially warm versus cold, reflective versus boorish, competent versus inept, earthy versus repressed, and benign versus mean-spirited humorous styles. The 10 styles were derived from the intercorrelations of 100 items depicting everyday humor behaviors as represented in thoughts, behaviors, and attitudes. The Humor Styles Questionnaire (HSQ; Martin et al., 2003) distinguishes four trait-like humor styles, namely, the affiliative, self-enhancing, aggressive, and self-defeating humor styles. The former two are considered to be adaptive and the latter two maladaptive functions of humor. In both instruments, the “humor styles” do not represent elementary flavors, types, or distinctive qualities of humor (such as sarcasm or nonsense) but compounds of humor behaviors (that go together) or humor functions at a more general level of abstraction. None of them relates to a “known” or established category of humor, but they represent new constructs that cluster more diverse humor behaviors or functions.

The present approach aims at supplementing the existing styles by investigating lower-level styles, namely *humor, fun, nonsense, wit, irony, satire, sarcasm*, and *cynicism* as described by Schmidt-Hidding (1963). We argue that these styles reflect established categories of humor (in the broad sense) and that they are narrower than the ones in the HBQD and HSQ, which allows for a more fine-grained differentiation of humor-related styles. For example, in the HBQD, sarcasm is one element of the mean-spirited humor style (that also involves wit to keep people at distance), and nonsense is part of the benign humor style (which also includes appreciating intellectual word play and regularly exchanging topical jokes). Also, the aggressive humor style in the HSQ does not allow distinctions between different forms of mockery, such as satire, sarcasm, and cynicism (see Ruch and Heintz, 2016a). Employing narrower but distinct styles also allows speaking of “using” a humor style, as these represent smaller units that can be enacted, trained, and modified more easily. Such a list is now sought after.

### Selecting and Describing the List of Comic Styles

The present manuscript represents the first step in a larger endeavor aimed at eventually arriving at a comprehensive list of lower-level styles, their classification, reliable and valid assessment, the study of their origin and consequences, life-span development, and the development and evaluation of interventions controlling (i.e., increasing, decreasing, or modifying) their use. We selected a manageable list from prior work in psychology, esthetics, philosophy, and other disciplines. In literary studies, humor styles (or: comic styles^1^) already have a longer tradition, and this crystalized knowledge can be transferred to the domain of assessment of individual differences. Different authors introduced various lists of styles. For example, Lauer (1974) distinguished nine styles; namely, humor, self-irony, comic in a narrow sense, fun, wit, irony, satire, sarcasm, and cynicism. Milner Davis (2003) identified a very comprehensive list covering farce/slapstick or low comedy, comedy of manners/wit or high comedy, romantic/festive or sentimental comedy (e.g., sitcoms), ironic/parodic or burlesque comedy, nonsense humor/absurdist comedy, “sick”/disgust comedy (e.g., U.S. “shock-u” comics), satire/satirical comedy, “black”/gallows/existential comedy, and tragicomedy. By perusing the table of index words of different handbooks and encyclopedias of humor (e.g., Raskin, 2008; Attardo, 2014; Wirth, 2017) a few more candidates could be added, especially if different disciplines, countries, etc. are involved. Yet it is also apparent that a smaller set of styles is more frequently mentioned, and those that have clear behavioral implications (i.e., that can be applied to distinguish individuals) can be selected for psychological investigation.

The work of Schmidt-Hidding (1963) helps bridging the gap between the comic styles rooted in literary studies of humor and a personality perspective interested in describing individual differences regarding humor use (for a detailed account of his approach, see Ruch and Heintz, 2016b). Schmidt-Hidding described eight styles with seven features (exemplified with sarcasm); namely, (1) intention, goal (to hurt the partner), (2) object (the corrupt world), (3) attitude of the agent as subject (e.g., derisive, feels like an undiscovered genius, thus often maliciously critical), (4) behavior toward others (e.g., hostile), (5) the ideal audience (e.g., subordinate and dependent people, who don’t dare to disagree), (6) method (e.g., ruthless exposure), and (7) linguistic peculiarities (e.g., ironic, with emphasis). Some of these features can be clearly located in the person, and others provide valuable additions to the definition of the constructs. For example, behavior is motivated by the intentions or goals which might be either conscious or not. The “object” might define individuals, as this is what they adhere to or find important. The attitude of the agent clearly is trait-like as is the behavior toward others. The ideal audience might be significant as people might search for the right audience to use their comic style. The last two features are less central to personality, but the use of these methods might be indicative of personality and individuals may or may not use the linguistic peculiarities well. Taken together, these seven features allow creating distinct prototypes of styles, and we supplemented these accounts by the study of other sources, and finally developed descriptions of the eight comic styles.

The prototypes of the styles, crystallized mainly from Schmidt-Hidding’s descriptions, can be described as follows. Four styles may be considered dark ones (as opposed to lighter ones; see below), as they constitute a family of mockery/ridicule. In particular, *sarcasm* aims at hurting others. The sarcastic person is described, among others, as being hostile and derisive and as using ruthless exposure to highlight the corrupt world. The ideal audience consists of subordinate and dependent people. High scorers would see themselves as malignant and critical when decrying the corruption, depravity, vice, or evil. They are prone to scorn and *schadenfreude*. *Cynicism* is aimed at devaluing commonly recognized values. Cynics exhibit a negative and destructive attitude. They use disillusionment and mockery to highlight weaknesses in the world. Cynics do not lack moral values in general, yet they disdain certain common norms and moral concepts and find them ridiculous. *Satire* (a.k.a. corrective humor; Ruch and Heintz, 2016b) shares with sarcasm and cynicism the detection of weaknesses and is aggressive. However, this is paired with attempts at goodness. This involves not only deprecating the bad and foolish, but also the intention of improving the world and correcting fellow humans. A satirist takes the ethical world as a measure of the real one and attempts to improve conditions by disclosing the true circumstances. The satirist is critical, often negative, tense and superior, but prefers the world to be moral and uses ridicule to better the world. Although the aggressive tendency is the common element, the mockery is not done on the basis of sheer pleasure, but it is grounded in a moral-based criticism. People with a critical mindset typically approve satire. The goodness of satire appeals to change inappropriate behaviors or mindsets without seriously damaging the interpersonal relations. *Irony*, as expressed in interactions, aims at creating a mutual sense of superiority toward others by saying things differently than they mean it. It does not entail lying as one assumes that smart people will understand what was actually meant irrespective of what was said. Ironic people are courting and letting in the intelligent, thereby at the same time mocking the stupid. Irony is a means of confusing the non-insiders and finding out who is a knowledgeable informed insider. Others may see them as conceited, superior, and frequently negative-critical.

There are lighter styles that do not contain these skeptic elements. They are very diverse despite sharing a more positive basis of interpersonal cooperation, benevolence, positive emotions, and cognitive capabilities. Specifically, *fun* (joking, jesting) is aimed at spreading good mood and good comradeship. People using this comic style are considered to be social, jovial, and also agreeable. In everyday life situations, they use teasing (waggish, impish) with friends and people accustomed to bawdy matters. They might see themselves as funny jokers and like to make mischievous jests. They play harmless tricks on friends and like to jest and act clownish. Next, *humor* (a.k.a. benevolent humor; Ruch and Heintz, 2016b) aims at arousing sympathy and an understanding for the incongruities of life, the imperfections of the world, the shortcomings of fellow humans, and the own mishaps and blunders. People with humor are realistic observers of human weaknesses, but treat them benevolently, often including themselves in the judgment rather than directing it exclusively at others. There is an understanding for humanity in all weaknesses, which are observed and shared with a jovial, relaxed, and contemplative audience. Humor comes “from the heart” and reflects a tolerant, loving attitude toward others that includes accepting their shortcomings. A person with humor in this sense knows that, both on a large and small scale, the world is not perfect. Still, with a humorous outlook on the world even the adversities of life can be amusing and be smiled at. A person using this comic style manages to arouse understanding and sympathy for imperfections and the human condition through humor. *Nonsense*, as intellectual and playful, cheerful fun, aims at exposing the ridiculousness of the sheer sense, though basically without any purpose. People enjoying nonsense describe themselves as playful and cheerful. They let their mind play, for example, by being creative with language and by playing with sense and nonsense. For them, incongruities do not need to be resolved, but the opposite holds true; that is, the more absurd, and the funnier. They create an upside-down world, use language in its imperfection, and find bizarre and fantastic stories amusing.

Finally, one style can be seen as part of the lighter styles despite also containing elements characteristic of the darker styles. *Wit* intends to illuminate like a flashlight, typically with a surprising punch line that uses unusual combinations created on the spot. A person using wit plays with words and thoughts, and they might be callous, malicious, and generally without sympathy for the “victims” in order to maximize the funny impact. Producing wit requires skills: It entails quickly reading situations and nailing non-obvious matters to the point in a funny way. They surprise others with funny remarks and accurate judgments of current issues, which occur to them spontaneously. They make relationships between disconnected ideas or thoughts and thus create a comical effect quickly and pointedly. Witty people might be tense, vain, and take themselves seriously, and look for an educated society that appreciates brief pointed utterances as an ideal audience.

### Considerations on the Structure of the Styles

The use of many narrower styles will yield interrelated scales, and the intercorrelations could be used to derive fewer (and more) abstract styles, which poses the question what these different levels are good for. To uphold the use of the narrower styles, it is important to demonstrate (a) that they can be separated conceptually and empirically and (b) that each style predicts different phenomena and is not redundant. Some individuals might “use” certain styles more often than others, but each style is still functionally different. Being sarcastic does not necessarily mean that one is also more cynical, although these two styles will be highly correlated. Training to be witty might enhance wit, but not necessarily satire, although both might correlate as well. This suggests that it is best to keep the concepts at this level of abstraction, rather than, for example, cluster them together and use aggregated styles.

**FIGURE 1:**
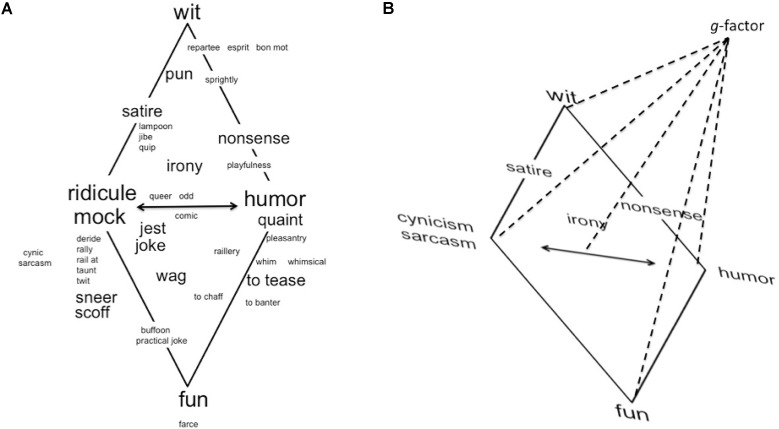
The eight comic styles in a schematic representation together with the key terms and other humor-related words (**A**, left) and in an individual-differences model (**B**, right). Adapted from Schmidt-Hidding (1963, p. 48).

However, one can look at the interrelations among the styles (based on covariations of individual differences in a sample) and conduct a second-order factor analysis for two reasons. First, one can examine how these interrelations can be represented in a smaller space and describe the styles at an aggregated level. While there is no intention of reducing these styles to a fewer number of concepts, it might provide insights into the structure of the styles and indicate where they overlap. Should styles correlate too highly, one might consider dropping some or combining them at a conceptual level to form a new scale (but not a factor derived from it). Second, structure-building methods could be applied to empirically test the assumptions of different authors about the structure inherent in this list of styles. For example, Lauer (1974) ordered the styles (in the sequence listed above) to reflect different mixtures of two tendencies, namely self-assertion (as a consciousness-limiting tendency) and participation (as a consciousness-expanding tendency). Humor assumes a special role in this model, as it allows for an optimum of “euphoric” self-assertion and participation, while cynicism is lowest in this respect. This allows for predictions about the relative proximity of these two styles as well as the postulate that two factors might be sufficient to represent most of the variance. Interestingly, Schmidt-Hidding (1963) ordered the styles similarly. However, these are spread along a rhomboid that is marked by what he considered to be key terms (i.e., the most frequent terms) in the field of the comic, namely humor, wit, fun, and mock/ridicule. These and some satellite words (with lower frequency) as well as the comic styles are depicted in a topographical model (see **Figure 1A**). While the generation of the model is not fully explicated and it is also not clear whether these terms would be still the most frequent nowadays, this configuration can be taken allowing for hypotheses about the structure of the eight comic styles to be tested empirically (Study 1). Furthermore, Schmidt-Hidding (1963) saw “energizing forces” behind the key terms and the satellite words. Accordingly, humor can be contrasted from the other three key terms as being based on a “sympathetic heart” (guided by love), not a “superior spirit” (like wit), moral critique, or even haughtiness (guided by hatred) like mock/ridicule, or vitality/high spirits (like fun). These descriptions allow deriving the hypothesis that unique predictors for comic styles may come from the domains of ability (for wit) and character (for the virtuous forms) in addition to traditional personality traits (Study 2).

Two types of testing the structure of comic styles seem appropriate; namely, the relation among the depicted key terms/comic styles (see **Figure 1A**) and one that considers individual differences in the comic styles (see **Figure 1B**). The former test can be based on a multidimensional scaling of a matrix representing the degree of similarity or dissimilarity between all comic styles or all terms listed (based on either judging similarities/dissimilarities directly, or computing them from raw scores) or based on a second-order factor analysis of ipsative data (eliminating the third dimension; i.e., individual differences). Then one can examine whether humor and mock/ridicule (represented by sarcasm and cynicism) are indeed opposing each other, in as much as love and hate are opposites. There is also a north–south distinction, but it is not seen as a bipolar dimension in **Figure 1A**. There is intellectual wit in the north marking more clever, hidden, and verbal comic creations, with satellite words connecting toward mock/ridicule (e.g., satire, jibe, quip and lampoon) and humor (e.g., nonsense, playfulness). Fun is in the south position, with satellite terms connecting to mock (e.g., sneer, scoff) and humor (e.g., tease, banter). While this is not a bipolar dimension, the former is more akin to high comedy and the latter to low comedy. The order of the eight styles can be examined as well. From the arrangement of terms, cynicism and sarcasm are expected to be the most difficult to distinguish as they are very close to each other. Comparing self- and other-reports helps to show whether these comic styles can actually be separated from one another (discriminant validity) and whether one’s self-evaluation and the perceptions of others converge (convergent validity).

The second type of testing the structure of comic styles involves a second-order factor analysis of individual differences in the use of comic styles, and somewhat different results may be expected due to the inclusion of the third dimension that represents a general factor (g-factor) of comic styles use (see **Figure 1B**). For example, while mock and humor are opposite as concepts in **Figure 1B** (i.e., implying a negative relationship), some individuals might engage in both and others in neither of them (i.e., suggesting even a positive relationship). This variance overlies the pattern of relations among the styles and alters the size (and potentially even sign) of the correlations. While there are people that clearly prefer mock over humor (and others that prefer humor over mock), this might happen at different levels of comic style use. Thus, by controlling the level, an initially perfect negative relation might turn into a slightly positive one. **Figure 1B** posits that the relations depicted in the rhombus (**Figure 1A**) only exist if the third dimension is kept constant (i.e., when individual differences do not occur or matter). Prior work with two different sets of preliminary markers for the eight comic styles (documented in Ruch, 2012) suggested that two or three second-order factors might be sufficient to represent the eight styles.

## Study 1

### Aims of Study 1

The overarching aim of Study 1 is to design and evaluate marker items for the eight comic styles (the Comic Style Markers, CSM) that can be used for both self- and other-reports, that represent the comic styles as identified in literary studies, and that allow measuring differences among individuals. In detail, this entails (a) confirming the item-level factor structure, (b) selecting suitable marker items (based on factor loadings and item statistics), (c) examining the reliability (internal consistency) and retest reliability of the CSM, (d) replicating the psychometric properties in a different language (English), (e) examining whether there is convergent and discriminant validity in self-other agreement, (f) determining socio-demographic correlates, and (g) examining the structure of the comic styles by looking at their intercorrelations, and vertical and hierarchical configurations (by means of hierarchical and ipsative second-order factor analysis).

### Methods^2^

#### Participants

Overall,^2^ five samples were employed in Study 1 (see **Table 1**). Sample 1 was used to select the best items from the pilot version of the CSM for the final version. Sample 2 was employed to test whether the final item selection could be replicated in an independent sample. Sample 3 investigated the test-retest reliability of the CSM after 1–2 weeks. This sample partially overlaps with another study in which everyday humor behaviors and the HSQ were investigated (Heintz, 2017b). Sample 4 investigated the self-other agreement by having two close others rate the participants on an other-report form of the CSM. This sample partially overlaps with another study in which the construct validity of the HSQ was investigated (Heintz, 2017a). Sample 5 investigated the English version of the CSM.

**Table 1:** Overview of the samples including basic descriptive statistics, measures, and analyses of Studies 1 and 2.

Samples	Gender	Age	Education	Nationality	Measures	Analyses
	*(M/F)*	*M* (*SD*)				
**Study 1**					
Sample 1			62.4% university-entrance diploma	69.3% German	CSM	Reliability, CITC
(*N* = 550)	26.7%/	25.04	29.3% university degree	14.5% Swiss	(pilot version)	Unidimensionality, EFA
	73.1%	(6.06)		11.3% Austrian		
Sample 2			41.7% university degree	44.0% German	CSM	Reliability, CITC
(*N* = 468)	33.3%/	37.15	38.0% university-entrance diploma	31.2% Austrian	(pilot version)	Unidimensionality, EFA
	65.6%	(15.11)		21.4% Swiss		
Sample 3			48.0% university students	65.5% Swiss	CSM	Test-retest reliability
(*N* = 148)	32.4%/	27.77	25.0% university-entrance diploma	25.7% German		CFA
	67.6%	(11.32)	23.0% university degree			
Sample 4			59.5% university students	83.3% Swiss	CSM	Self-other agreement
(*N* = 210)	26.7%/	26.35	17.6% university entrance diploma	10.5% German	CSM	CFA
	73.3%	(11.02)	16.2% university degree		(other- report form)	
Sample 5			–	38.6% American	CSM	Reliability, CITC
(*N* = 303)	33.0%/	29.77		30.7% British	(English adaptation)	Unidimensionality, CFA
	67.0%	(14.76)				
**Study 2**					
Sample 1			51.0% university-entrance diploma	57.3% German	CSM	Partial correlations and
(*N* = 999)	30.1%/	30.77	35.3% university degree	20.3% Austrian	MRS-25	multiple regressions
	69.9%	(12.81)		18.0% Swiss		
Sample 2	23.8%/	39.24	36.9% school or university students	51.8% Swiss	CSM	Partial correlations and
(*N* = 252)	76.2%	(16.52)		42.3% German	VIA-IS	multiple regressions
Sample 3			100% university students	-	CSM	Partial correlations and
(*N* = 214)	20.7%/	24.13			I-S-T 2000 R	multiple regressions
	79.3%	(3.88)			MSEI	

*CSM, Comic Style Markers; MRS-25, Inventory of Minimal Redundant Scales; VIA-IS, VIA Inventory of Strengths; I-S-T 2000 R, Intelligence Structure Test 2000 Revised; MSEI, measure for self-estimated intelligence; CITC, corrected item-total correlation; EFA, exploratory factor analysis; CFA, confirmatory factor analysis*.

#### Instruments

A pilot version of the CSM was generated, which was designed to mark the comic styles fun, humor, nonsense, wit, irony, satire, sarcasm, and cynicism (Schmidt-Hidding, 1963) as clearly as possible. The pilot version of the CSM comprised 73 marker items that depict the eight comic styles. Descriptions of the styles were compiled incorporating the elements discussed by Schmidt-Hidding (1963) and supplemented by other sources, such as descriptions of the comic styles in the literature, encyclopedias, dictionaries, and so on. Special care was taken that these elements could be related to individuals and eventually be transformed into corresponding items. This was achieved by studying definitions of the styles and transforming them into statements depicting everyday thoughts, feelings, and actions, while taking care of sticking to the definitions as purely as possible. A seven-point Likert format (1 = “strongly disagree” to 7 = “strongly agree”) was utilized. There were between 6 and 13 marker items per comic style in the pilot version. Sample items are “I quickly read situations and can nail non-obvious matters to the point in a funny way” (wit) and “I accept the imperfection of human beings and my everyday life often gives me the opportunity to smile benevolently about it” (humor).

The revised version of the CSM includes 48 marker items, with six marker items for each comic style. The same seven-point Likert format is utilized. The items are listed in the Electronic Supplementary Material (Supplementary Table S1). For now, the main aim was to preserve the meaning of the styles and to be able to study the concepts. A final questionnaire to measure the comic styles comprehensively (e.g., by adding other relevant styles, facets of styles, and additional descriptions of the comic styles) will be developed at a later point in time.

The other-report version consists of the same 48 marker items as the CSM. The only difference is that the marker items were rephrased to capture other-reports. Specifically, pronouns and verb forms were adapted, and “I” was replaced by the participant’s first name. It employs the same seven-point Likert scale. The English version of the CSM was adapted in a translation back-translation procedure. Inconsistencies were jointly resolved in a group discussion among the first, second, and third author of this paper.

#### Procedure

The five samples were collected online via www.surveymonkey.com (Samples 1, 2, and 5) or www.unipark.info (Samples 3 and 4). Other variables were collected that are not relevant for the present study. The study was conducted in compliance with the local ethical guidelines and participants provided online informed consent. In Sample 3, participants completed the final version of the CSM twice in a period of 1–2 weeks. In Sample 4, participants were provided with a link to an online survey including the other-reports of the CSM, which they forwarded to two close others.

#### Analyses

The rationally derived 73 items of the pilot version of the CSM (listed in the Supplementary Table S2) were subjected to three analyses to select the final items: Descriptive item analyses, corrected item-total correlations (CITC), and loadings on the first unrotated principal component (FUPC) to ensure the unidimensionality of the scales. These CITC and FUPC analyses were refined in two rounds, as the item pool influences the outcomes of these analyses. The analyses were conducted in Sample 1 and then replicated in Sample 2.

To examine the factor structure of the revised version of the CSM, both exploratory and confirmatory factor analyses were conducted. Because these item-level analyses require large sample sizes, the exploratory and confirmatory factor analyses were conducted in pooled samples (Samples 3 + 4, and Samples 1 + 2, respectively). The exploratory factor analysis (EFA) was a principal axis factoring with oblimin rotation, as the factors were expected to be dependent (conducted with SPSS 20). The confirmatory factor analysis (CFA) was estimated with the *lavaan* package (Rosseel, 2012) in *R* (R Core Team, 2015). The MLR estimator was employed to yield robust standard errors, and the factors were allowed to correlate with each other. Fit indices were evaluated by the recommendations for acceptable fit of Schermelleh-Engel et al. (2003): comparative fit index (CFI) ≥ 0.95, root mean square error of approximation (RMSEA) ≤ 0.08 with a confidence interval close to the RMSEA, and standardized root mean square residual (SRMR) ≤ 0.10. For the CFI somewhat lower values were expected in the present analysis due to the large number of variables per factor, which can lead to low CFI values even if the model is correctly specified (see Kenny and McCoach, 2003).

To investigate test–retest reliability, the scores from the first assessment of the CSM were correlated with the scores from the second assessment (Sample 3). In Sample 4, self-other convergence (convergent validity) was tested by correlating the self-reports of the CSM with the aggregated other-reports (aggregated across two raters per participant).

### Results

#### Identification of Markers: Reduction of Items

First, the descriptive statistics of the 73 marker items in the pilot version of the CSM were analyzed. The distribution of the items should approximate a normal distribution, so items that were skewed or kurtotic (values > |2|) were removed (2 items from the irony scale). Second, the CITC of the items were computed and compared to the correlations of the items with the other seven scales. Similarly, the FUPC of the items belonging to one scale was extracted in a principal component analysis, and the factor score was saved. Then the 73 marker items were correlated with each of the eight factor scores. These two steps should ensure that (a) each marker item relates to the scale/factor it belongs to, and (b) each marker item relates more strongly to the scale/factor it should belong to than to the other scales/factors. This procedure contributes to the reliability (internal consistency and unidimensionality) and factorial validity of the resulting scales. The marker items should have CITC of ≥0.30 (see Traub, 1994) and a loading on the FUPC of ≥0.40 (see Stevens, 2012). Also, the correlations of the marker item with the other scales should be at least 0.05 lower than the CITC, and the correlations of the marker items with the other factors should be at least 0.10 lower than the loading on the FUPC. Based on these criteria, 15 items were deleted (0–5 items from each scale), resulting in a second pilot pool of 56 items. In the second round, the remaining items were investigated with similar CITC and FUPC analyses. This resulted in an exclusion of 8 additional marker items (0–5 items from each scale), resulting in 48 marker items (six marker items per comic style). Importantly, the marker items that were excluded in Sample 1 were also those that showed the lowest CITC and loadings on the FUPC in Sample 2, replicating the selection of the revised 48 marker items (i.e., the CSM).

#### Reliability and Factor Structure of the Comic Styles

**Table 2** shows the psychometric properties of the revised set of marker items of the CSM in the pooled construction and replication samples (Samples 1 and 2). As shown in **Table 2**, the psychometric properties supported the reliability of the eight scales. Internal consistencies ranged from 0.66 (humor) to 0.89 (cynicism), with most values being > 0.80. The CITCs ranged from 0.33–76, indicating that the marker items related to their scales, yet they were not redundant. Homogeneity (or unidimensionality) was supported in CFAs, indicated by high loadings on the latent factor (all > 0.40) and by mostly acceptable model fits, ranging from χ^2^_(9)_ = 46–129 (*p*s < 0.001), CFI = 0.92–0.96, RMSEA = 0.06–0.12 (90% confidence intervals [0.05–0.10, 0.08–0.13], and SRMR = 0.03–0.05. Supplementary Table S3 additionally shows the descriptive statistics of the CSM in all samples.

**Table 2:** Descriptive statistics, reliability, factor structure, and test–retest correlations of the Comic Style Markers (CSM) in the German-speaking samples.

CSM	*M*^a^	*SD*^a^	α^a^	CITC^a^	Homogeneity^a^	EFA^b^	CFA^a^	*r*_tt_^c^
Fun	4.37	1.16	0.86	0.57–0.70	0.62–0.77	0.51–0.75	0.62–0.81	0.88
Humor	5.01	0.83	0.66	0.33–0.49	0.41–0.66	0.20–0.67	0.45–0.58	0.74
Nonsense	4.93	1.09	0.85	0.58–0.71	0.62–0.79	0.37–0.85	0.65–0.78	0.89
Wit	4.80	1.06	0.87	0.61–0.71	0.50–0.81	0.56–0.80	0.67–0.77	0.89
Irony	4.46	1.17	0.82	0.47–0.72	0.65–0.77	0.37–0.71	0.52–0.82	0.86
Satire	4.22	1.02	0.75	0.41–0.58	0.47–0.70	0.31–0.64	0.47–0.67	0.78
Sarcasm	3.65	1.33	0.85	0.50–0.76	0.55–0.86	0.43–0.65	0.55–0.85	0.83
Cynicism	3.55	1.38	0.89	0.67–0.74	0.71–0.80	0.36–0.74	0.72–0.80	0.88

*CITC, range of the corrected item-total correlations; Homogeneity, range of the loadings on the latent factor (separate for each comic style); EFA, exploratory factor analysis (principal axis factoring with oblimin rotation, loadings on the expected factors); CFA, confirmatory factor analysis (range of the loadings on the latent factors); *r*_tt_, test–retest reliability across 1–2 weeks. ^a^Results from pooled Samples 1 and 2 (*N* = 826–1018). ^b^Results from pooled Samples 3 and 4 (*N* = 358). ^c^Results from Sample 3 (*N* = 148)*.

In the EFA, the eight factors explained 58.2% of the total variance (eigenvalues 1.18–10.93, rotated sums of squared loadings 3.63–5.62). While the scree test indicated the retention of either four or six factors, the parallel analysis suggested the retention of nine factors, and the revised minimum average partial test suggested the retention of seven factors. However, we decided to extract eight factors for the following reasons: (a) we theoretically expected eight factors, (b) the factor loadings were more clearly interpretable compared to the other solutions, (c) the communalities were mostly high (range = 0.13–0.70, *Mdn* = 0.51), (d) the items always loaded highly on their intended factors (ranging from 0.20 to 0.75; see **Table 2**), and (e) these loadings were always higher than the loadings on any of the other factors (maximum |0.50|). Only one humor item (“I am a realistic observer of human weaknesses, and my good-natured humor treats them benevolently”) loaded negatively on sarcasm (-0.27) and positively on satire (0.27), which was slightly higher than the loading on humor (0.25).

The CFA model indicated a mostly acceptable fit: χ^2^_(1052)_ = 3310 (*p* < 0.001), CFI = 0.86, RMSEA = 0.05 (90% confidence interval [0.049,0.053], and SRMR = 0.07). Loadings were high for each factor, ranging from 0.45 to 0.85 (see **Table 2**). Finally, the test-retest reliability was high for all scales (0.74–0.89, *Mdn* = 0.87). As the time interval was rather short (1–2 weeks), this indicates at least short-term stability of the eight scales.

The intercorrelations of the eight scales ranged from essentially 0 to 0.67 (sarcasm and cynicism), with a median correlation of 0.37 (pooled Samples 1 + 2, *N* = 1,018). The zero correlations suggest that there will be no general factor in the field of the comic. However, it should be mentioned that the zero correlations all either involved sarcasm or cynicism and hence the other comic styles showed a positive manifold (i.e., only positive intercorrelations). The factor correlations were similar to the scale intercorrelations. In the factor analyses, the factor correlations were highest between sarcasm and cynicism (0.44 in the EFA and 0.81 in the CFA) with a median correlation of 0.21 (EFA) and 0.45 (CFA). As the CFA correlations were true-score correlations, this supports the notion that sarcasm and cynicism were similar, yet not interchangeable.

#### English Version of the Comic Style Markers

**Table 3** shows the descriptive statistics, reliability, and factor structure of the CSM in the English-speaking sample. The reliabilities of the comic styles were also sufficient, ranging from 0.79 (irony) to 0.88 (wit and satire). The expected factor structure was supported in a CFA, which showed a mostly acceptable model fit: χ^2^_(1′052)_ = 2′005 (*p* < 0.001), CFI = 0.86, RMSEA = 0.06 (90% confidence interval [0.05,0.06], and SRMR = 0.07). Loadings were high for each factor, ranging from 0.49 to 0.85. Homogeneity of the factors was also supported, indicated by high loadings (>0.40) and by mostly acceptable model fits, ranging from χ^2^(9) = 16–49 (*p*s < 0.07), CFI = 0.92–0.99, RMSEA = 0.05–0.12 (90% confidence intervals [0.01–0.09,0.08–0.15], and SRMR = 0.03–0.05). Also Tucker’s phi was computed, which indicates the factor congruence between the eight EFA factors from the German- and English-speaking samples. The nonsense scale could be considered equal across both languages, while fair similarity was obtained for fun, humor, and wit (and to some degree for irony, satire, and cynicism). A lack of similarity was only obtained for sarcasm. Tucker’s phi at the item level indicated sufficient similarity for four of the six items (>0.84). Two items (“I am a sharp-tongued detractor” and “My laughter is occasionally derisive and expresses schadenfreude”) showed lower loadings on the sarcasm factor and higher loadings on wit and satire, resulting in low convergence (0.64 and 0.15, respectively). Thus, similarity between the English- and the German-speaking samples was sufficient for all comic styles except for two sarcasm items.

**Table 3:** Descriptive statistics, reliability, and factor structure of the Comic Style Markers in the English-speaking sample.

Comic styles	*M*	*SD*	α	CITC	Homogeneity	CFA	Tucker’s Phi
Fun	4.91	1.24	0.85	0.54–0.70	0.61–0.79	0.60–0.79	0.91
Humor	5.14	1.01	0.82	0.46–0.71	0.47–0.85	0.49–0.85	0.90
Nonsense	5.11	1.09	0.86	0.60–0.68	0.65–0.75	0.68–0.73	0.95
Wit	5.06	1.15	0.88	0.61–0.78	0.59–0.74	0.66–0.80	0.93
Irony	4.53	1.09	0.79	0.49–0.63	0.65–0.84	0.56–0.71	0.80
Satire	4.07	1.27	0.88	0.67–0.73	0.72–0.79	0.72–0.80	0.84
Sarcasm	3.91	1.34	0.87	0.56–0.76	0.58–0.85	0.61–0.82	0.70
Cynicism	3.94	1.24	0.84	0.55–0.69	0.59–0.79	0.65–0.75	0.83

*N = 303. CITC, range of the corrected item-total correlations; Homogeneity, range of the loadings on the latent factor separate for each comic style; CFA, confirmatory factor analysis (range of the loadings on the latent factors)*.

The intercorrelations among the comic styles were slightly higher than in the German-speaking samples, ranging from small positive correlations to 0.74 (sarcasm and cynicism), with a median correlation of 0.49. In the CFA, the factor correlations were highest between sarcasm and cynicism (0.84) with a median correlation of 0.56.

### Demographic Differences in the Comic Styles

Next, it is of interest whether the comic styles differed across several demographic variables. **Table 4** shows the correlations and analyses of covariance of the CSM with the demographic variables. Gender and age showed several meaningful correlations with the CSM. While most of them were small and significant due to the large sample size, a few noteworthy and theoretically expected relationships emerged. First, men tended to score higher in all comic styles than women (except for humor), with the strongest effects found for cynicism, satire, and sarcasm. This is in line with the more mocking and critical nature of these comic styles. Regarding age, humor and, to a lesser extent, nonsense tended to be shown more often by older than by younger people. Conversely, younger people engaged more often in irony, sarcasm, and cynicism than older people. When considering the love vs. hate dimension underlying the comic styles, the more love-related comic styles tended to increase with age, while the more hate-related ones tended to decrease with age.

**Table 4:** Demographic differences in the Comic Style Markers.

	Correlations		*F*-values of ANCOVA (age and gender as covariates)			
Comic styles	Gender	Age	Education^1^	Nation^2^	Family^3^	Housing^4^
Fun	-0.08^∗∗^	-0.07^∗^	0.49	1.71	1.50	4.02^∗∗^
Humor	-0.03	0.19^∗∗∗^	2.01	1.38	1.30	3.88^∗∗^
Nonsense	-0.09^∗∗^	0.10^∗∗^	1.20	4.61^∗^	2.67^∗^	1.81
Wit	-0.13^∗∗∗^	0.08^∗∗^	3.10^∗^	1.88	1.16	1.98
Irony	-0.14^∗∗∗^	-0.23^∗∗∗^	2.54^∗^	0.54	1.71	0.64
Satire	-0.20^∗∗∗^	-0.05	0.59	0.32	1.26	1.88
Sarcasm	-0.17^∗∗∗^	-0.13^∗∗∗^	1.43	0.33	0.44	1.30
Cynicism	-0.27^∗∗∗^	-0.12^∗∗∗^	1.27	0.67	2.59	2.26^∗^

*N = 1’013. ANCOVA, analysis of covariance. Gender was coded as 1 = male, 2 = female. Spearman’s rank correlations were used for education and for age. ^∗^*p* < 0.05; ^∗∗^*p* < 0.01; ^∗∗∗^*p* < 0.001. ^1^Five education categories: <10 years (*n* = 15), apprenticeship (*n* = 93), university entrance diploma (*n* = 520), university degree (*n* = 354), and doctoral degree (*n* = 25). ^2^Three nation categories: Germany (*n* = 579), Switzerland (*n* = 168), and Austria (*n* = 207). ^3^Four family categories: single (*n* = 486), in a relationship (*n* = 310), married or registered relationship (*n* = 156), and divorced or widowed (*n* = 46). ^4^Six housing categories: living alone (*n* = 274), living with partner (*n* = 225), living with partner and children (*n* = 100), living with children (*n* = 18), living in a shared apartment (*n* = 251), and living with parents/relatives (*n* = 135)*.

The education level made a difference regarding wit ($\eta_{p}^{2}$ = 0.012) and irony ($\eta_{p}^{2}$ = 0.010). Follow-up pairwise comparisons (Bonferroni-corrected) showed that people who held a doctoral degree scored significantly higher on wit (*M* = 5.48, *SD* = 0.70) than those with an apprenticeship (*M* = 4.64, *SD* = 1.16; *p* = 0.010, *d* = 0.78), while no pairwise comparison was significant for irony. The three nations showed significant differences only in nonsense ($\eta_{p}^{2}$ = 0.010); that is, Austrians (*M* = 5.15, *SD* = 0.99) scored higher than Germans (*M* = 4.82, *SD* = 1.13; *p* = 0.008, *d* = 0.30). The family situation showed a significant difference in nonsense ($\eta_{p}^{2}$ = 0.008); that is, those in a relationship (*M* = 4.94, *SD* = 1.07) scored higher than those who were divorced or widowed (*M* = 4.71, *SD* = 1.09; *p* = 0.037, *d* = 0.21). The housing situation made a difference regarding fun ($\eta_{p}^{2}$ = 0.020), humor ($\eta_{p}^{2}$ = 0.019), and cynicism ($\eta_{p}^{2}$ = 0.011). Those who lived in a shared apartment scored significantly higher on fun (*M* = 4.61, *SD* = 1.16) and on humor (*M* = 5.05, *SD* = 0.77) than those living alone (*M* = 4.22, *SD* = 1.21, and *M* = 4.89, *SD* = 0.95; *p* = 0.008, *d* = 0.33, and *p* = 0.004, *d* = 0.18 respectively), while no pairwise comparison were significant for cynicism. Overall, a few meaningful, but small demographic differences emerged. The only large effect was found for wit, which was influenced by the level of education of the participants.

#### Construct Validity-I: Self-Other Convergence

**Table 5** shows the convergent and discriminant correlations of the self- and other-reports of the CSM. The convergent correlations were large for each comic style (ranging from 0.44–0.56, *Mdn* = 0.50), supporting the convergent validity of the CSM. Importantly, the convergent correlations were always larger than the discriminant correlations (both regarding the median and maximum discriminant correlations). This also supports the discriminant validity of the CSM.

**Table 5:** Convergent and discriminant correlations of self-reports and other-reports (averaged across two close others) of the Comic Style Markers.

		Discriminant correlations		
Comic styles	Convergent	Median	Minimum	Maximum
Fun	0.55^∗∗∗^	0.17	0.04	0.33
Humor	0.44^∗∗∗^	0.13	-0.12	0.27
Nonsense	0.44^∗∗∗^	0.16	0.11	0.33
Wit	0.56^∗∗∗^	0.19	0.08	0.36
Irony	0.49^∗∗∗^	0.25	-0.04	0.37
Satire	0.40^∗∗∗^	0.20	0.11	0.32
Sarcasm	0.50^∗∗∗^	0.17	-0.12	0.40
Cynicism	0.52^∗∗∗^	0.15	-0.12	0.40

*N = 210; ^∗∗∗^*p* < 0.001*.

#### Construct Validity-II: Structure of the Comic Styles

##### Intercorrelations of the scales

**Table 6** shows the intercorrelations among the eight comic styles. As in Samples 1 and 2, the correlations between humor and satire and cynicism were close to zero, and correlations were largest among sarcasm and cynicism. No negative correlations were found among any of the comic styles.

**Table 6:** Intercorrelations among the eight Comic Style Markers.

Comic styles	Fun	Humor	Nonsense	Wit	Irony	Satire	Sarcasm
Humor	0.33^∗∗∗^						
Nonsense	0.44^∗∗∗^	0.38^∗∗∗^					
Wit	0.40^∗∗∗^	0.46^∗∗∗^	0.28^∗∗∗^				
Irony	0.31^∗∗∗^	0.16^∗∗∗^	0.24^∗∗∗^	0.34^∗∗∗^			
Satire	0.28^∗∗∗^	0.39^∗∗∗^	0.25^∗∗∗^	0.36^∗∗∗^	0.46^∗∗∗^		
Sarcasm	0.19^∗∗∗^	0.00	0.19^∗∗∗^	0.18^∗∗∗^	0.53^∗∗∗^	0.46^∗∗∗^	
Cynicism	0.15^∗∗^	0.03	0.28^∗∗∗^	0.18^∗∗∗^	0.45^∗∗∗^	0.53^∗∗∗^	0.69^∗∗∗^

*Samples 3 + 4 (*N* = 358); ^∗∗^*p* < 0.01; ^∗∗∗^*p* < 0.001*.

##### Second-order factor analyses

**FIGURE 2:**
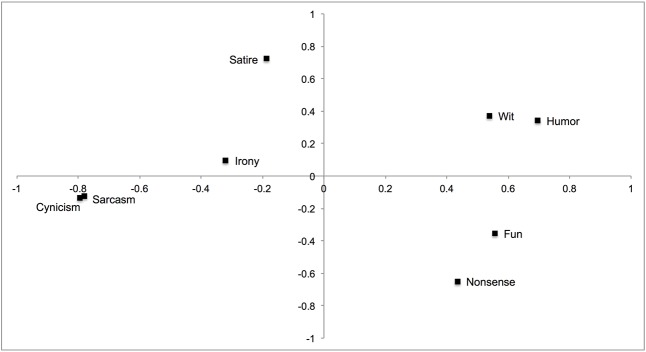
Plot of factor loadings of the ipsative comic styles (based on principal components analyses).

First, an analysis of the ipsative scores was conducted (principal components analysis). For each individual the mean across the eight styles was computed and subtracted from the eight scores. This way every individual had the same mean (but the standard deviations could vary). The scree test indicated two factors, which are displayed in **Figure 2**. The configuration was similar to **Figure 1A**, with a few peculiarities. Regarding the darker styles, sarcasm and cynicism were closely together and irony and satire are close to where wit was. On the lighter side, the arrangement of fun, humor and wit were as expected, yet nonsense was closer to fun, rather than between humor and wit. Finally, the main axis separated the lighter and darker styles, which is not as salient in **Figure 1A**, but still suggests that this was the major bipolar dimension in the comic styles.

Second, a PCA was performed on the normative data of the eight comic styles for Samples 3 and 4. The scree test suggested the extraction of two or three factors (first four eigenvalues: 3.49, 1.59, 0.77, 0.60, 0.50). Therefore all solutions between a FUPC and four oblique factors were studied. Thus, a hierarchical factor analysis (Goldberg, 2006) was conducted (see **Table 7**).

**Table 7:** Factor pattern (oblimin rotation) of principal components analyses based on the intercorrelations among the eight comic styles.

		Two-factor solution		Three-factor solution		
Comic styles	FUPC	Factor 1	Factor 2	Factor 1	Factor 2	Factor 3
Fun	**0.58**	0.05	**0.70**	0.23	**0.44**	**0.79**
Humor	**0.49**	-0.19	**0.85**	0.05	**0.84**	**0.42**
Nonsense	**0.57**	0.11	**0.62**	0.26	0.28	**0.88**
Wit	**0.61**	0.07	**0.71**	0.27	**0.80**	0.36
Irony	**0.71**	**0.67**	0.18	**0.72**	0.35	0.30
Satire	**0.75**	**0.59**	0.33	**0.69**	**0.61**	0.20
Sarcasm	**0.68**	**0.92**	-0.13	**0.88**	0.08	0.20
Cynicism	**0.68**	**0.90**	-0.09	**0.87**	0.10	0.23
Correlation with F1			0.31		0.23	0.23
Correlation with F2						0.37

*N = 358. FUPC, first unrotated principal component. Loadings > |0.40| in bold*.

The eight styles all loaded on the first unrotated factor (explaining 43.6% of the variance) and then split up in the positively correlated light and dark styles at step two. While both factors were largely unipolar, there was a tendency for humor to load negatively on the dark styles factor and sarcasm to load negatively on the light styles factor. The three-factor solution had the lighter styles split up into two, and a four-factor solution was clearly an overextraction, as specific factors emerged. Thus, the three-factor solution (explaining 73.2% of the variance) was selected for interpretation. Factor 1 (tentatively labeled “mockery, ridicule”) was highly loaded by sarcasm and cynicism as well as by satire (i.e., morally based ridicule) and irony (i.e., a technique that may be used for ridicule). The common element was that people mock and ridicule in a funny way. The second factor (“good humor”) was primarily loaded by wit, humor and satire, but also to a lower degree by fun and irony. The commonality was that they are the more competent and even virtuous comic styles. The loading of satire was due to the moral goodness that is merged with mockery when ridicule is done to better a situation. The third factor (“enjoyment of humor”) was primarily loaded by two scales, namely nonsense and fun, and slightly also by humor. This factor was definitely underdefined and needs more markers for a precise interpretation in the future.

### Discussion

The main aim of Study 1 was accomplished, namely to design and validate a set of marker items that represent the eight comic styles based on the descriptions derived from literary studies (the CSM). The descriptions were useful for formulating marker items and the scales got refined in a first empirical analysis. Six marker items proved to be adequate to measure the styles with sufficient reliability (i.e., internal consistency, unidimensionality/homogeneity, and test–retest reliability). Regarding validity, the factorial validity of the items was established by CFAs; that is, the marker items measured the styles they were intended to measure. Furthermore, the self-other correspondence was sufficiently high, and it was even possible to distinguish between sarcasm and cynicism. The self-other correspondence with a median of 0.50 was much higher than for the earlier one-item measure (Ruch, 2012) and in the range typical for personality instruments. While most analyses were done with a German-speaking sample, the first testing of an English version proved successful too. Most importantly, discriminant validity was supported; that is, all styles (including cynicism and sarcasm) could be distinguished from each other. Thus, for now the CSM can be recommended for use in future studies. Once more styles are identified and once items from the experiential world of laypeople supplement these prototypes, a final instrument, the *Comic Styles Profiler*, will be introduced.

Thus, the eight styles were conceptually and empirically different. Nevertheless, some styles were more similar to each other than others, and when eliminating individual differences (i.e., a g-factor) through ipsatizing the scores, the proposed bipolarity of mockery styles (sarcasm, cynicism) and good-natured humor was verified. Furthermore, cynicism and sarcasm were close to each other with satire (as a moral critique) and irony also having a higher proximity to wit. On the light side, the (vertical) order of wit, humor and fun was also found, with the exception that nonsense was not located between humor and wit, but close to fun. It remains to be studied whether the location of nonsense was due to emphasizing the fun element in the enjoyment of nonsense in the marker items or whether this was simply the more appropriate location not anticipated by the more intuitive model (depicted **Figure 1A**), which was not based on measurement.

However, when individual differences were allowed for, there was no strict opposition of light and dark styles (as some individuals might be high or low in both), but they rather defined the first unrotated factor together. They then tended to fall into a lighter and darker cluster, and the former ones fell into a shallower (non-serious cheerfulness) and a more profound (resourceful) subgroup. All these factors intercorrelated uniformly positive, suggesting that only one factor (i.e., the g-factor as depicted in **Figure 1B**) was needed to account for the intercorrelations. Such a hierarchical model (i.e., entailing eight lower order styles, three style factors, and a general factor) is possible; however, it will need to be built on more variables helping to identify the factors more clearly (see also Supplementary Figure S1 for a schematic representation of this model). Mockery (or “laughing at”) is a factor that emerged in the present study as well as in previous studies with preliminary measures (Ruch, 2012). Mockery combines all dark styles, with cynicism and sarcasm being at its core, and satire and irony having high but not pure loadings. Schmidt-Hidding (1963) suggested that the use of these styles implies having malicious, mean-spirited goals and attitudes, intentions of hurting other people and demonstrating superiority. Therefore, using this set of comic styles will hurt or upset others. Still, there are nuances in this factor, and future research needs to study these styles further and also examine their relation to katagelasticism (i.e., the joy of laughing at others; Ruch et al., 2014) and to the aggressive (Martin et al., 2003) and mean-spirited (Craik et al., 1996) humor styles (see Ruch and Heintz, 2016a, for a preliminary investigation of the overlap of these different conceptualizations of humor styles).

This was different for the light styles, which have different goals, but typically go along with positive affect. They came in two clusters, a more basic enjoyment of humor factor and a more profound good humor factor. The former can be seen as enjoyment of the non-seriousness in communication and social interaction; it is more socio-affective and refers indulging in playing pranks, clowning around, good-natured kidding, brightening others up, indulging in gibberish talk, and playing with meaning, sense and nonsense. This factor will be more similar to the socially warm and boorish humor styles (Craik et al., 1996) and the hilarity component of cheerfulness, namely the facets of low threshold for smiling and laughter, a broad range of active elicitors of cheerfulness and smiling/laughter, and a generally cheerful interaction style (Ruch et al., 1996). Also, this factor is expected to predict enjoyment of various forms of humor stimuli, including non-sophisticated forms and low comedy. The good humor factor is marked by the more profound styles of humor, wit, and also satire, which, taken together, entail more cognitive efforts (i.e., mindfully observing incongruities in daily lives), resilience when facing adversity (ability to see the funny side in adversities or short-comings), and a general aiming at the good. It is more related to the cheerful composedness of trait cheerfulness (Ruch et al., 1996), the self-enhancing humor style (Martin et al., 2003), and the reflective and benign humor styles (Craik et al., 1996), without being identical with any of these. There are more resources needed for this style, like mindfully detecting the incongruities in life, the capacity to describe them, and the relaxedness to deal with them in a lighthearted way. While both forms of light styles represent cheerfulness, the former might be also related to low seriousness and the latter might be related to a robustness of mood (i.e., low bad mood) and character strengths.

## Study 2: Construct Validity

Comic styles are trait-like, either as typical behavior (i.e., temperament/personality), maximal performance (i.e., ability), or, more recently, morally valued traits (i.e., character). Humor instruments have been studied mostly in relation to personality (as represented, for example, by the five-factor model, FFM; see McCrae and Costa, 2013) for a long while now (see Ruch, 2008, for an overview of studies). Liking to laugh, entertaining others, telling jokes, and experiencing positive emotions are part of components of extraversion, and hence we expect the light styles to be correlated positively with extraversion. Neuroticism represents a disposition to negative emotions and worry, and thus we expect it to relate negatively to humor in face of adversity (i.e., being able to laugh at oneself, to cope with stress) and humor performance (e.g., wit). Agreeableness vs. antagonism determines the tone toward others; that is, cooperative and friendly vs. critical or hostile. In line with this, we expect agreeableness to be negatively related to the dark styles (especially sarcasm and cynicism) and positively related to the benevolent treatment of shortcomings (i.e., humor). Openness to experience (or culture, intellect) provides the capacity for generating humor, and we expect it to correlate with wit, but also the other styles involving a production of humor. Conscientiousness refers to components like order, dutifulness, and self-deliberation, but also low spontaneity. Hence it is difficult to imagine conscientiousness being positively related to any particular style, and we do not make specific predictions for conscientiousness. In sum, to the extent that positive and low negative emotions, imagination and friendliness vs. antagonism are involved in a comic style, we expect it to show a correlation with extraversion, emotional stability, culture/openness to experience, and agreeableness, respectively.

Ability is maximal performance, and in humor (in the broad sense), there are a few components that represent ability in processing, creating, and delivering humor. For example, producing funny punch lines on the spot will require verbal ability. Perceiving incongruities and combining them in a witty statement requires mental capacities as well. Hence, it is not surprising that humor and ability were rarely studied together except for wit or humor production. Nevertheless, there are studies showing that people of higher intelligence displayed a higher appreciation of nonsense (Terry and Ertel, 1974; Wierzbicki and Young, 1978; Hehl and Ruch, 1985). Taken together, we expect that mostly wit has some relation with intelligence, and of the different components of intelligence it is verbal (but not numerical or figural) intelligence that displays the highest coefficients.

More recently, character has been introduced to the study of personality through the postulate of character being composed of virtues, character strengths, and situational themes (Peterson and Seligman, 2004). Virtues are seen as the core characteristics valued by moral philosophers and religious thinkers, and character strengths are the psychological ingredients—processes or mechanisms—that define the virtues, or distinguishable routes to displaying one or another of the virtues. Factor analyses of the strengths often reveal five factors, namely emotional, interpersonal, intellectual and theological strengths, as well as strengths of restraint (Peterson and Seligman, 2004).

We expect several links between the comic styles and this model of character. First, what is laughable and what is not laughable has historically been shaped by virtues (Ruch, 1998) and hence positive (as well as negative) correlations are expected between some comic styles and virtues. In detail, strengths molded by humanity may relate positively to humor (and negatively to the mockery styles), the cognitive strengths defining wisdom and knowledge might relate to wit, and temperance (e.g., prudence, self regulation) and transcendence (e.g., gratitude, spirituality) strengths suggest lower engagement in mockery.

Second, as humor is one of the 24 strengths, it is interesting to see what contents went into the definition and how the scale relates to the eight comic styles. For Aristotle (Chase, 1890), the virtuous form of humor (i.e., the ready-witted form) is to joke and amuse without hurting. For Aristotle, “ready wit” is moderation in the desire to amuse others, and the excess desire is buffoonery (amuse others too often, striving for laughter at all costs, laughing excessively, relentless mockery), and the deficient desire is boorishness (e.g., not getting involved in joking at all, feeling negatively about it). Similarly, Peterson and Seligman (2004, p. 530) define the humorous person as someone “skilled at laughing and teasing, at bringing smiles to the faces of others, at seeing the light side, and at making (not necessarily telling) jokes.” They note that in the domain of humor, some forms are mean (e.g., mockery, ridicule, sarcasm) or on the border (e.g., parody, practical jokes), and they only include forms that “serve some moral good—by making the human condition more bearable by drawing attention to its contradictions, by sustaining good cheer in the face of despair, by building social bonds, and by lubricating social interaction” (Peterson and Seligman, 2004, p. 530). This suggests stronger overlaps between humor as character strength and fun, humor and wit, and zero conceptual overlap with sarcasm and cynicism. Additionally, satire forms something morally good (i.e., correcting wrongdoings with the aim to better society or people) but also involves some criticism, which might result in lower correlations. Thus, we expect satire and the mockery styles (sarcasm, cynicism) to differentially relate to the strengths and virtues; when controlling for the mockery element (by partialling out sarcasm and cynicism), we anticipate satire to more strongly positively relate to strengths and virtues. Low correlations are expected for irony, which may involve criticizing through a compliment or state something positive through negative words. Taken together, we anticipate the correlations between the comic styles and humor as character strength to differ between highly positive to virtually zero.

Study 2 aims at extending the construct validity investigations of the CSM. Construct validity is examined by studying the relation between comic styles and more general traits of personality, character, and ability. Special attention will be given to examine humor as character strength.

### Methods^3^

#### Participants

An overview^3^ of the samples of Study 2 is given in **Table 1**. Sample 1 consisted of subsets of Samples 1 and 2 from Study 1 that also completed a personality measure. Sample 2 completed the CSM and a measure of character strengths. This sample overlaps with another study in which the comic styles and subjective well-being were investigated (Ruch et al., 2018). Sample 3 completed the CSM and measures of intelligence (self-reported and psychometrically tested).

#### Instruments

*The Inventory of Minimal Redundant Scales* (*MRS-25*; Schallberger and Venetz, 1999) lists 25 pairs of bipolar adjectives for the assessment of the Big Five personality dimensions *extraversion*, *agreeableness*, *conscientiousness*, *emotional stability*, and *culture*. The answers format is a bipolar six-point scale. In the present sample, internal consistencies were satisfactory, ranging from α = 0.76 (agreeableness and culture) to 0.87 (conscientiousness).

The *VIA Inventory of Strengths* (VIA-IS; Peterson et al., 2005; German adaptation by Ruch et al., 2010) is a 240-item questionnaire for the assessment of the 24 character strengths (10 items per strength) covered by the VIA classification (Peterson and Seligman, 2004). It employs a 5-point Likert-style scale ranging from 1 (“very much unlike me”) to 5 (“very much like me”). A sample item is “I never quit a task before it is done” (persistence). Internal consistencies in the present sample ranged from α = 0.68 (honesty) to 0.91 (religiousness) with a median of 0.78. To obtain aggregated scores a principal component analysis of the VIA-IS scales with subsequent varimax rotation of five factors was conducted. The five-factor solution closely resembled the solution reported in Ruch et al. (2010), with Tucker’s Phi coefficients being 0.95 (emotional), 0.90 (interpersonal), 0.88 (intellectual), 0.98 (theological), and 0.95 (restraint).

The *Intelligence Structure Test Revised* (I-S-T 2000 R; Amthauer et al., 2001) consists of nine subtests. It allows the assessment of fluid as well as crystallized intelligence. For the present study, subtests for verbal (analogies), numerical (arithmetical tasks), and spatial (cube tasks) intelligence were used. The tests for verbal, numeric, and spatial intelligence were taken together as a total score of intelligence. All tests are speed tests; i.e., the administration was timed. The I-S-T 2000 R is widely used and well established in the German-speaking countries. Norm scores were computed according to German age and gender norms (*M* = 100, *SD* = 10).

*Measure for self-estimated intelligence* (MSEI; Proyer and Ruch, 2009). Participants had to rate their ability on a line from “low” to “high ability” for the domains of verbal, numeric, and spatial intelligence. Each position on the scale ranging from lowest to highest self-estimated ability may be marked (on a scale from 0 “lowest ability” to 100 “highest ability”). A total score was computed from all self-estimations as a general self-estimated ability score. The single dimensions were explained by a short sentence [e.g., “verbal: Dealing with language and words (e.g., eloquence)”].

#### Procedure

For Samples 1 and 2, all questionnaires were presented online. Participants were recruited via different channels; for example, social media, mailing lists, or newspaper articles containing the link to the respective study. All participants provided consent and participated voluntarily. Sample 3 consisted of students at the University of Zurich who were attending a lecture on psychological assessment and the data was collected in paper–pencil format for this study. All studies were performed in accordance with the local ethical guidelines and online or written informed consent was supplied.

#### Analyses

To assess the overlap between the criteria and the comic styles, correlations were computed. Since the measures showed small but consistent correlations with age and gender, these variables were controlled for in partial correlations (Supplementary Table S4 also shows the zero-order correlations as well as the descriptive statistics of all measures). Furthermore, standard multiple regressions were computed to assess how much variance could be explained in total in each of the comic styles and in each of the criteria.

### Results

#### Personality

The correlations between the FFM traits (MRS-25) and the eight comic styles were computed and are presented in **Table 8** (controlling for age and gender). Multiple correlations with the FFM traits as criteria showed that the variance in extraversion, agreeableness, culture and emotional stability was well explained by comic styles, while conscientiousness had a significant, but low contribution. Likewise, multiple correlations computed for the comic styles as criteria showed that wit and humor were most potently predicted, followed by fun, sarcasm, and cynicism, and eventually nonsense, satire, and irony.

**Table 8:** Partial correlations and multiple regressions of the comic styles with the Inventory of Minimal Redundant Scales (controlled for age and gender).

Personality traits	Fun	Humor	Non.	Wit	Irony	Satire	Sarc.	Cyn.	*R*/Adj. *R*^2^
Extraversion	0.42^∗∗∗^	0.31^∗∗∗^	0.14^∗∗∗^	0.37^∗∗∗^	0.06^∗^	0.16^∗∗∗^	-0.04	-0.09^∗∗^	0.49/0.23
Agreeableness	0.04	0.25^∗∗∗^	0.05	0.00	-0.17^∗∗∗^	-0.11^∗∗∗^	-0.41^∗∗∗^	-0.32^∗∗∗^	0.49/0.24
Conscientiousness	-0.13^∗∗∗^	-0.09^∗∗^	-0.15^∗∗∗^	-0.08^∗^	-0.04	-0.11^∗∗∗^	-0.10^∗∗^	-0.13^∗∗∗^	0.21/0.04
Emotional stability	0.19^∗∗∗^	0.35^∗∗∗^	0.13^∗∗∗^	0.30^∗∗∗^	0.03	0.09^∗^	-0.10^∗^	-0.02	0.40/0.16
Culture	0.24^∗∗∗^	0.34^∗∗∗^	0.28^∗∗∗^	0.40^∗∗∗^	0.08^∗^	0.21^∗∗∗^	-0.03	0.04	0.47/0.22
*R*	0.44	0.51	0.33	0.53	0.21	0.29	0.42	0.36	
Adj. *R*^2^	0.19	0.25	0.11	0.27	0.04	0.09	0.17	0.13	

*N = 999. Non., nonsense; Sarc., sarcasm; Cyn., cynicism; Adj., adjusted. ^∗^*p* < 0.05; ^∗∗^*p* < 0.01; ^∗∗∗^*p* < 0.001*.

**Table 8** shows that each of the comic styles had a unique pattern of correlations with personality. In more detail, the prime correlation of sarcasm and cynicism was low agreeableness and to a smaller extent low conscientiousness. Additionally, cynics tended to be introverted and sarcastic individuals tended to be emotionally instable. Satire and humor both correlated with all FFM traits, but there were also differences. While satire (like the two styles of mockery and irony) correlated negatively with agreeableness, humor yielded a positive correlation. Furthermore, the positive correlations with extraversion, culture, and emotional stability were numerically higher for humor than for satire. Wit, fun and nonsense shared the predictors of humor, except for agreeableness; however, regarding fun, extraversion was the best predictor, and regarding both wit and nonsense, culture yielded the highest coefficients.

Interestingly, in this sample humor was uncorrelated with both cynicism and sarcasm, and hence it is unlikely that a third variable correlated with them with a different sign (as agreeableness did with humor and the mockery styles). To establish bipolarity between the love vs. hate comic styles (see **Figure 1A**), an index was computed by subtracting the average of sarcasm and cynicism from humor. This index correlated positively with agreeableness (*r* = 0.48), thus suggesting that agreeableness vs. antagonism as a personality dimension was aligned with humor-related benevolence vs. mockery. Removing individual differences enhanced the correlations that were found for the individual styles. Other high multiple correlations also showed bipolarity in the predictors, however, again with a predominance of one side, namely extraversion (cynics were introverted) and emotional stability (sarcasm was on the neuroticism side).

#### Character Strengths

The correlations between the five strengths factors (VIA-IS) and the eight comic styles (controlled for age and gender) were computed and are presented in **Table 9**. The correlations between the 24 characters strengths and the eight comic styles (controlled for age and gender, as well) are depicted in Supplementary Table S5.

**Table 9:** Partial correlations and multiple regressions between the character strengths factors (derived from the VIA-Inventory of Strengths) and the Comic Style Markers (controlled for age and gender).

Strength factors	Fun	Humor	Non.	Wit	Irony	Satire	Sarc.	Cyn.	*R/*adj. *R^2^*
Emotional	0.41^∗∗∗^	0.40^∗∗∗^	0.25^∗∗∗^	0.47^∗∗∗^	0.24^∗∗∗^	0.23^∗∗∗^	0.17^∗∗^	0.04	0.56/0.29
Interpersonal	0.13^∗^	0.12	-0.02	0.02	-0.18^∗∗^	-0.07	-0.26^∗∗∗^	-0.27^∗∗∗^	0.43/0.16
Restraint	-0.26^∗∗∗^	-0.11	-0.18^∗∗^	-0.14^∗^	-0.04	-0.13^∗^	-0.15^∗^	-0.13^∗^	0.34/0.09
Intellectual	0.10	0.22^∗∗∗^	0.27^∗∗∗^	0.46^∗∗∗^	0.23^∗∗∗^	0.27^∗∗∗^	0.13^∗^	0.23^∗∗∗^	0.55/0.30
Theological	0.16^∗^	0.08	0.08	-0.01	-0.11	-0.02	-0.28^∗∗∗^	-0.21^∗∗^	0.46/0.18
*R*	0.53	0.49	0.44	0.70	0.48	0.44	0.56	0.53	
Adj. *R*^2^	0.27	0.23	0.18	0.47	0.21	0.18	0.30	0.27	

*N = 252. Non., nonsense; Sarc., sarcasm; Cyn., cynicism; adj., adjusted. ^∗^*p* < 0.05; ^∗∗^*p* < 0.01; ^∗∗∗^*p* < 0.001*.

**Table 9** shows that each of the strengths factors was involved in the prediction of comic styles and each comic style was predicted by character strengths, and there were positive as well as negative coefficients. In detail, emotional strengths (loaded by zest, hope, bravery, but also humor) predicted fun, wit, and humor well, were still significant for nonsense, irony, satire, and sarcasm, and were uncorrelated with cynicism. Interpersonal strengths (loaded by fairness, teamwork, kindness, leadership, forgiveness) predicted fun positively and sarcasm, cynicism, and irony negatively. Strengths of restraint (loaded by prudence, humility, self-regulation, persistence) tended to go along with low scores in most comic styles, in particular with fun. Intellectual strengths (loaded by love of learning, creativity, open-mindedness but also appreciation of beauty and excellence) strongly predicted wit, and (albeit less strongly) other comic styles with a cognitive emphasis, namely, nonsense, humor and irony. Only fun was uncorrelated with intellectual strengths. Theological strengths (loaded by religiousness, gratitude, and appreciation of beauty and excellence) positively predicted fun and correlated negatively with sarcasm and cynicism.

Most interestingly, Supplementary Table S5 shows that the VIA-IS humor scale correlated significantly positively with every comic style, but to a different extent. Fun (*r* = 0.63), wit (*r* = 0.61), and humor (*r* = 0.58), had high coefficients, followed by nonsense (*r* = 0.38), satire (*r* = 0.39), and irony (*r* = 0.33, all *p*s < 0.001). Sarcasm (*r* = 0.15) and cynicism (*r* = 0.13, *p* < 0.05) had small but significant zero-order correlations. However, a multiple regression analysis predicting the VIA-IS humor scale yielded significant and positive beta weights only for fun (β = 0.41, *p* < 0.001), wit (β = 0.30, *p* < 001), humor (β = 0.25, *p* < 001) and satire (β = 0.14, *p* = 0.027), and negative ones for sarcasm and cynicism (β = -0.17, *p* = 0.011 each).

Next the assumption was tested that in satire two elements blend, namely mockery of someone combined with a good intention. Satire, or corrective humor, is not decrying something foolish or immoral for malicious pleasure, but for changing things to the better, leaving the good relationship intact. To highlight the good character element in satire, partial correlations were computed between the five strengths factors and satire, controlling for age and gender, but also for sarcasm and cynicism. Satire, bereft of the critical tone, was exclusively positively related to character, namely emotional strengths (*r* = 0.24), interpersonal strengths (*r* = 0.23), intellectual strengths (*r* = 0.26), and theological strengths (*r* = 0.21, all *p*s < 0.001). The factor describing strengths of restraint (*r* = -0.06) had no significant correlation, as it was a constant in all styles. Altogether 18 of the 24 strengths yielded significant positive correlations, underscoring the involvement of good character in corrective humor (see Supplementary Table S5).

#### Ability

The correlations between intelligence (based on a test and self-reports) and the eight comic styles (controlled for age and gender) were computed and are presented in **Table 10**.

**Table 10:** Partial correlations of the Comic Style Markers with self-rated and measured intelligence (controlled for age and gender).

Intelligence	Fun	Humor	Nonsense	Wit	Irony	Satire	Sarcasm	Cynicism
**Measured**					
Verbal	0.06	0.08	0.05	0.19^∗∗^	0.09	-0.04	0.08	-0.03
Numerical	0.01	0.11	-0.07	-0.03	0.08	0.09	-0.02	0.02
Spatial	-0.07	-0.03	0.01	-0.08	0.08	-0.01	-0.07	0.06
Total	-0.03	0.06	-0.02	0.04	0.12	0.06	0.00	0.03
**Self-rated**					
Verbal	0.10	0.28^∗∗∗^	0.08	0.48^∗∗∗^	0.01	0.12	0.04	-0.03
Numerical	-0.01	0.08	0.04	0.01	0.13	0.04	0.04	0.09
Spatial	-0.02	0.05	0.07	0.05	0.17^∗^	0.06	-0.09	0.08
Total	0.04	0.21^∗∗^	0.13^∗^	0.28^∗∗∗^	0.17^∗^	0.16^∗^	0.02	0.14^∗^

*N = 214 (self-rated intelligence, assessed with the Measure for self-estimated intelligence) and N = 199 (measured intelligence, assessed with the Intelligence Structure Test 2000 Revised). ^∗^*p* < 0.05; ^∗∗^*p* < 0.01; ^∗∗∗^*p* < 0.001*.

As expected, the correlation between measured verbal intelligence and wit was indeed positive and significant, and it was the only significant correlation. The coefficient was rather low, but considering that ability and personality are rarely related, the size of the coefficient was as expected.

Interestingly, and not surprisingly, there were more (and higher) correlations for self-rated intelligence. First, the correlation of self-rated verbal intelligence and wit was much higher, underscoring that using the same method (self-reports) yielded higher relationships than divergent methods (self-reports, test). However, there were also positive correlations with humor, albeit smaller than for wit. The total intelligence score correlated most strongly with wit, followed by humor. Additionally, there were also small correlations with nonsense, irony, and satire. Thus, in five of the styles, people who scored higher also assumed that they were higher in several of the intelligence scales (and the total score). The differences between measured and rated intelligence are plausible but also striking, underscoring that there is method variance involved. However, it is also clear that the use of wit is also based on a higher (measured) verbal intelligence.

### Discussion

Comic styles tap differently into personality, ability and character, and each of the comic styles had a unique set of predictors, underscoring the necessity to be separated. A person’s involvement in the ludicrous is an expression of one’s personality traits (including the valued traits) and selectively also verbal intelligence. Wit requires an astute mind that allows to quickly read situations and nailing non-obvious matters to the point in a funny way. Obviously, ability in the verbal (rather than numerical or figural) domain provides the link between measured intelligence and wit. Moreover, self-rated verbal ability, but also creativity (VIA-IS scale), cognitive strengths (as a strengths factor), and culture/intellect (MRS-25) assume the position to be especially predictive of wit. Future studies will need to show whether these predictors overlap, and whether performance measures of wit (e.g., being able to write witty punch lines to caption-removed cartoons) is predictive of wit in the present instrument. Thus, this component of humor (in the broad sense) can indeed be seen as also drawing on individual differences in ability, a domain neglected in humor research.

Character, the moral subdomain of personality, was demonstrated to be relevant as well, and the use of fine-grained measures of both character and humor allowed for a more comprehensive investigation. For once, the use of both sarcasm and cynicism was regulated by theological (e.g., gratitude, religiousness) and interpersonal (e.g., fairness, forgiveness) strengths (see also Beermann and Ruch, 2009; Müller and Ruch, 2011). These components of the good character counteracted a frequent expression of mockery, while cognitive strengths and emotional strengths (sarcasm only) favored it. Character was also involved in satire. While sarcasm and cynicism may be fueled by the joy of mockery without the involvement of a moral sense, in satire there are good intentions of correcting misdoings. Focusing on the motivation for corrections (i.e., removing mockery), the moral sense was revealed in nearly all factors of strengths (except restraint). Thus, the comparatively lower zero-order correlations for satire were the product of negative (i.e., pointing out flaws in others) and positive (i.e., moral justification for the criticism) tendencies. Wit and humor were most highly correlated with humor as character strength, and these comic styles were well predicted by the individual character strengths and by the strength factors. Humor had only positive character correlates both at the level of the individual strengths and the strength factors and was hence the best indicator of good character. Wit and fun had overwhelmingly positive correlates with character, but also a negative correlation with the strengths of restraint factor, which was based on individual strengths, namely, prudence (fun) and humility (wit). Agreeableness, just as emotional strengths (primarily loaded by strengths of humanity and courage), was also indicative of humor and negatively related to mockery. As there was no simultaneous assessment of personality and character, it cannot be decided whether and where character provides incremental predictions of the virtuous comic styles over personality.

There were distinct correlations of personality with comic styles, which fit well to predictions. Some strengths underlay all styles, some either the light or the dark, and other strengths additionally underlay some specific styles. Conscientiousness (like strengths of restraint) tended to yield low negative correlations with all styles, suggesting that this is a minor constant in engaging in humor at all. Extraversion, emotional stability, and culture correlated higher with the light styles than with the others. While low agreeableness was related to the dark styles (most strongly for sarcasm and cynicism) and negative affect related to sarcasm and cynicism, both were also linked inversely to humor, suggesting that these traits were sensitive to the motivational difference between laughing at and laughing with. Other traits also had humor on both sides of the dimension, namely extraversion (cynics were introverted), emotional stability (low in sarcasm) and antagonism (humor was agreeable). There was no style that involved low culture/openness.

Thus, in sum, the results provide indirect and at least partial support for the assumption that the comic styles reflect different domains of human functioning, with fun, humor, wit, and mock/ridicule reflecting forces of vitality/high spirits, a sympathetic heart, a superior spirit, and moral sense or haughtiness/maliciousness, respectively (depicted in **Figure 1A**). Specifically, cognitive strengths and verbal intelligence indicate a “superior mind”, agreeableness, emotional strengths and humanity reflect a “sympathetic heart,” zest and extraversion represent “vitality,” and low agreeableness represents “haughtiness.” There was no direct predictor for moral sense, and hence satire remains without a direct potent predictor. While these results confirm the lay psychologist view on humor and personality, future studies will emphasize the contemporary models of personality, character, and ability.

## Overall Discussion

Overall, the present studies represent a starting point for research defining and measuring more narrow styles of humor (in the broad sense), as they have been discussed for a long time in the literature, but have not yet been utilized in psychology. These styles represent a broad variety of humor and tap into personality and character as well as ability. These styles will be more easily “used,” trained, and modified than the existing ones in the literature. The overlap in the scales allows aggregating the styles to more general styles, which, in turn, potentially might form a general factor of humor. This analysis, again, might be the basis for forming a hierarchical model with three levels that needs to be completed and evaluated in future studies. Individual differences in humor might be described by the general level (concerning the overall humor potential of a person), by a profile in aggregated styles (informing about engagement in specific domains of humor), and by a profile in specific styles (that describes differences more fine-grained and is closest to behavior). These levels will be useful for different types of studies. For example, humor trainings best address the lower level; relations to health or work-related variables might be most parsimoniously studied at a midlevel (where the discovery of more general patterns will be sufficient). Again, if economy is important, the overall humor potential might be sufficient. However, the list of styles is not yet exhaustive and hence more research is needed in order to build a comprehensive model. This allows showing whether the assumption of a general factor is tenable or not. In particular, for a more complete description of the domain of humor, components of the ineptness in humor use or forms of humorlessness are needed.

As the present studies replicated the results found with prior markers (Ruch, 2012), the validity of the three-factor structure was substantiated. However, although the factors explained 70% of the variance, this is still lower than the reliability of the scales, and thus the scales had unique variance that gets lost when analyzing the factors only. Hence, the major level of analyses should still be the level of styles. Further research will show what the unique contributions of the individual styles are and where aggregation is meaningful. In an EEG study of 52 participants, potential brain mechanisms underlying different types of humor were investigated (Papousek et al., 2017). It provided evidence for the unique status of humor among the light styles, and the overlapping effects of sarcasm, cynicism and irony among the mocking comic styles. Specifically, phasic changes in the functional coupling of prefrontal and posterior cortex (EEG coherence) during other people’s auditory displays of happy (i.e., laughter) and sad mood (i.e., crying) were recorded and related to comic styles. The results support the view that typical comic styles develop in accordance with the rewarding values of their implicit outcomes (e.g., interaction partners are joyful or upset), which in turn reflect the individuals’ interpersonal goals. While there are four light comic styles, the results underscored that they were heterogeneous and that there was indeed only humor that had the “laughing with” quality. As in the structural analyses, the dark styles were more homogeneous, yet satire (i.e., the only dark style where the hurting aim might be diluted by the positive intentions in corrective humor) acts differently. Other studies also provided preliminary validation; for example, Ruch et al. (2018) provided evidence that the styles had different relations to well-being (e.g., wit, humor and fun correlated positively with life satisfaction, while cynicism correlated negatively). Further validation studies of the CSM will add knowledge to the uniqueness and common core of the different styles.

### Limitations

One obvious limitation is that in a first step the measurement of the styles was restricted, and there are other comic styles that could be considered for inclusion, such as black (gallows, sick) humor or absurd humor. These should be identified and examined in future studies to see if they entail elements that are not yet covered by the CSM. Furthermore, this approach will not lead to the description of the various forms of ineptness in humor, as these are typically not described in the literature. Hence, this is not a complete model of humor, and it needs to be supplemented by forms of humorlessness (e.g., Ruch and Hofmann, 2012; Ruch et al., 2014). Second, as the concepts of interest are complex by nature, they also require several elements to be present in the items to capture them adequately (e.g., “I am a realistic observer of human weaknesses, and my good-natured humor treats them benevolently”). Still, future research could develop different assessment strategies to tease apart these elements in separate items and test if they also mark the eight comic styles. Third, many of the findings were based on self-reports, and future studies should employ several assessment methods. Fourth, the samples employed were mostly well-educated, and thus replications with samples with a more varied educational background are needed.

## Conclusion

In two studies, we presented and tested the Comic Style Markers (CSM), a set of 48 marker items that represent individual differences in eight comic styles: Fun, humor, nonsense, wit, irony, satire, sarcasm, and cynicism. Both studies supported the construct validity of the CSM. Specifically, the eight comic styles were shown to be theoretically and empirically distinguishable and to relate to different outcomes (personality, character strengths, and intelligence). The CSM thus provides a starting point for more fine-grained investigations of humor-related styles, ultimately aiming at identifying a comprehensive list of narrow and specific comic styles that can be enacted, trained, and modified.

## Author Contributions

WR initiated the project and designed the concepts, all authors collected the data, SH, LW, and WR analyzed the data. All authors contributed to the writing of the manuscript, read it critically and gave consent to its publication.

## Conflict of Interest Statement

The authors declare that the research was conducted in the absence of any commercial or financial relationships that could be construed as a potential conflict of interest.

## Footnotes

1 When talking about “comic styles,” it becomes obvious that these authors adhere to a different terminological system that is used in some academic disciplines in some countries. As this is in conflict with contemporary English-speaking psychology, these two should be briefly contrasted as they assign different roles to the key term “humor.” The historical nomenclature in literature stems from the field of esthetics where the funny (or: the comic) — defined as the faculty of being able to make someone laugh or to amuse — is distinguished from other esthetic qualities, such as beauty, harmony, or the tragic. In this tradition humor is simply one element of the funny — as are wit, fun, nonsense, sarcasm, ridicule, satire, or irony, and humor is in opposition to them (e.g., humor and sarcasm are excluding each other). Humor is not a neutral term here but exclusively positive. The alternative, almost incompatible, current use of “humor” in contemporary psychological research is an umbrella term for all phenomena of the funny, including the capacity to perceive, interpret, and enjoy but also create and perform non-serious incongruous communications (for an overview, see Ruch, 2008). Obviously, in this terminology humor has replaced the comic/funny as the supreme term and is treated as a neutral concept; that is, humor is not restricted to positive occasions for laughter. From this perspective it is not a contradiction to speak about “sarcastic humor”; that is, sarcasm that is funny. In the present article, we stick to the notion of “comic styles” to mark their origin, knowing that they can as well be called “humor styles” when one works within the other frame of reference, which is most prevalent in contemporary psychology. We consider the operational definition important rather than the labels.

2 Data and materials of Study 1 can be obtained from the corresponding author upon request.

3 Data and materials of Study 2 can be obtained from the corresponding author upon request.
