# Supplementary material for: Broadening Humor: Comic Styles Differentially Tap into Temperament, Character, and Ability
Source: Front Psychol. 2018 Jan 18;9:6. doi: 10.3389/fpsyg.2018.00006 (PMC5778606; doi:10.3389/fpsyg.2018.00006)
Supplement: Supplementary file 1 [file Table_1.PDF]

Table S1.
Items of the Comic Style Markers

No.	Comic style	Marker items
01	Fun	I am a funny joker.
02	Irony	I can converse with close friends in a way that only we know what is meant, but outsiders don't sense that it is merely irony.
03	Wit	I have the ability to tell something witty and to the point.
04	Sarcasm	Biting mockery suits me.
05	Humor	I am a realistic observer of human weaknesses, and my good-natured humor treats them benevolently.
06	Satire	I have a critical attitude toward arrogant and unfair people and my mockery serves to establish equality and justice.
07	Nonsense	I like nonsensical humor.
08	Cynicism	I tend not to trust in the sincerity of some intentions and values and often unmask them by cynical remarks.
09	Fun	I like to make mischievous jests and thus spread a good mood in my group.
10	Irony	I enjoy it when someone says the opposite of what he/she means and expects that insiders will understand it and outsiders won't.
11	Wit	I quickly read situations and can nail non-obvious matters to the point in a funny way.
12	Sarcasm	I am a sharp-tongued detractor.
13	Humor	When my humor is aimed at human weaknesses, I include both myself and others.
14	Satire	I parody people's bad habits to fight the bad and foolish behavior.
15	Nonsense	I like humor when it aimlessly plays with sense and nonsense.
16	Cynicism	In general, human beings and the world are weak and I don't mind devaluating generally accepted values by cynical remarks.
17	Fun	I occasionally play harmless tricks on my friends and thus make them laugh.

18	Irony	My irony unveils who is smart enough and understands something and who does not.
19	Wit	I have a sharp wit and intellect and can tell stories with many punch lines.
20	Sarcasm	I have a bitter, biting kind of mockery at my disposal, which I express both directly and indirectly (e.g., by means of irony).
21	Humor	On a large and small scale, the world is not perfect, but with a humorous outlook on the world I can amuse myself at the adversities of life.
22	Satire	When fellow humans or institutions demonstrate their superiority unjustified, I use biting humor to belittle them.
23	Nonsense	Humor doesn't have to make sense; the opposite holds true for me: the more absurd, the funnier.
24	Cynicism	Human beings trust in values and morality too naïvely and blindly, which nourishes my ridicule and mockery.
25	Fun	I like to be clownish.
26	Irony	Whoever understands my irony is, along with me, superior to those who don't understand it.
27	Wit	I surprise others with funny remarks and accurate judgments of current issues, which occur to me spontaneously.
28	Sarcasm	I occasionally exhibit bitter scorn.
29	Humor	I accept the imperfection of human beings and my everyday life often gives me the opportunity to smile benevolently about it.
30	Satire	I caricature my fellow humans' wrongdoings in a funny way to gently urge them to change.
31	Nonsense	I find bizarre and fantastic stories amusing.
32	Cynicism	I disdain some moral norms and view them cynically, although I don't lack a sense of moral values in general.
33	Fun	I like to tease my friends in a funny way.
34	Irony	If I say something that is ironic, there is always someone in my group who understands it, and others who don't.

35	Wit	My wit and astute mind help me to be quick witted.
36	Sarcasm	I am often malignant and critical if I decry the corruption, depravity, vice, or evil.
37	Humor	Humor is suitable for arousing understanding and sympathy for imperfections and the human condition.
38	Satire	I like to ridicule moral badness to induce or increase a critical attitude in other people.
39	Nonsense	Absurdities amuse me.
40	Cynicism	I have a cynical attitude towards some common norms and moral concepts; I don't believe in them and mostly find them ridiculous.
41	Fun	I like to make jests and to be silly.
42	Irony	Our irony confuses those who don't understand it, as my close friends and I uphold what we really mean.
43	Wit	I can make relationships between disconnected ideas or thoughts and thus create quickly and pointedly a comical effect.
44	Sarcasm	My laughter is occasionally derisive and expresses schadenfreude.
45	Humor	Even when facing unpleasant events I can keep my distance and discover something amusing or funny in it.
46	Satire	If the circumstances are not as they actually should be, I poke fun at these moral transgressions or societal wrongdoings, hoping to improve them in the long term.
47	Nonsense	I like humor that flies in the face of logic.
48	Cynicism	I tend to show no reverence for certain moral concepts and ideals, but only scorn and derision.

Notes. Instructions: "In the following you will find a number of statements. These apply to the way you experience and respectively express humor. Please read each statement carefully and indicate to what extent the statements apply to you or not. Please answer spontaneously and honestly by selecting one out of seven answers."

Items are answered on a seven-point scale: 1 = strongly disagree, 2 = disagree, 3 = slightly disagree, 4 = neither disagree nor agree, 5 = slightly agree, 6 = agree, 7 = strongly agree. Scale scores are derived by computing the mean of the responses to the six items of each comic style.

Table S2.
Items of the Pilot Version of the Comic Style Markers (Original Items in German)

No.	Comic style	Item
01	Fun	Mein Alltag bietet oft Anlass für lustige Spässe.
02	Irony	Ich mag Ironie.
03	Wit	Ich habe oft witzige Einfälle, die den Nagel auf den Kopf treffen.
04	Sarcasm	Ich bin oft sarkastisch.
05*	Humor	Ich bin ein realistischer Beobachter menschlicher Schwächen, und mein gutmütiger Humor geht liebevoll mit diesen um.
06*	Satire	Ich habe eine kritische Haltung gegenüber überheblichen und ungerechten Menschen und mein Spott dient der Herstellung von Gleichheit und Gerechtigkeit.
07*	Nonsense	Ich mag Nonsens-Humor.
08	Cynicism	Ich empfinde manche bürgerliche Normen und Moralvorstellungen als lächerlich und kommentiere sie dann bissig oder zynisch.
09*	Fun	Ich bin ein lustiger Spassvogel.
10	Irony	Ich finde es lustig, einen Sachverhalt durch sein Gegenteil auszudrücken.
11*	Wit	Ich habe die Fähigkeit, etwas treffend und geistreich erzählen zu können.
12*	Sarcasm	Beissender Spott liegt mir.
13*	Humor	Wenn mein Humor auf menschliche Schwächen zielt, dann meine ich mich und andere gleichermassen.
14*	Satire	Ich parodiere die Unarten von Menschen, um so das Schlechte und Törichte zu bekämpfen.
15*	Nonsense	Ich mag Humor, wenn er zweckfrei mit Sinn und Unsinn spielt.
16	Cynicism	Ich habe eine zynische Einstellung bzw. Haltung.
17*	Fun	Ich mache gerne spitzbubenhafte Scherze und verbreite so gute Laune in meiner Gruppe.

18*	Irony	Ich kann mich mit meinen Vertrauten so unterhalten, dass nur wir wissen was gemeint ist, Aussenstehende aber nicht ahnen, dass es bloss Ironie ist.
19	Cynicism	Ich habe ein gesundes Misstrauen, wenn Leute „gute“ Absichten und Ziele vor sich her tragen und mache mich darüber lustig.
20*	Wit	Ich habe einen scharfen Witz und Verstand und kann Geschichten mit vielen Pointen erzählen.
21*	Sarcasm	Ich bin ein scharfzüngiger Lästerer.
22*	Humor	Die Welt im Grossen und Kleinen ist nicht perfekt, aber durch eine humorige Sicht der Welt kann ich mich auch an Widrigkeiten des Lebens erheitern.
23*	Satire	Wenn Mitmenschen oder Institutionen ihre Überlegenheit ungerechtfertigt ausspielen, setze ich bissigen Humor ein, um sie kleiner zu machen.
24*	Nonsense	Humor muss keinen Sinn machen; im Gegenteil, für mich gilt: je absurder, desto lustiger.
25	Irony	Ich mag es, wenn jemand Ironie benutzt; ich finde es schnell raus, wenn jemand Dinge anders sagt als er oder sie es meint.
26	Cynicism	Ich glaube, dass viele Leute sich was vor machen und mein Spott hilft, sie zu desillusionieren und ihnen ihre falschen Hoffnungen zu nehmen.
27*	Fun	Ich spiele meinen Freunden gelegentlich harmlose Streiche und bringe sie so zum Lachen.
28	Irony	Ich verstelle mich gerne und lasse dann aber durch Signale (z.B. Mimik, Gestik, Betonung, Anführungszeichen) den Zuhörer erkennen, dass ich das Gesagte nicht wörtlich, sondern ironisch verstanden wissen will.
29	Cynicism	Ich habe Glauben und Vertrauen in die Menschheit aufgegeben und finde, dass Zynismus weiteren Enttäuschungen vorbeugt.
30*	Wit	Ich habe eine rasche Auffassungsgabe und kann nicht offensichtliche Dinge auf witzige Art auf den Punkt bringen.
31	Sarcasm	Es macht mir Freude, das Korrupte und Schlechte in der Welt rücksichtslos blosszustellen bzw. lächerlich zu machen.
32*	Humor	Ich akzeptiere die Unvollkommenheit der Menschen und mein Alltag bietet mir oft Anlass, darüber wohlwollend zu schmunzeln.

33*	Satire	Ich karikiere Fehlverhalten meiner Mitmenschen auf lustige Weise, um sie damit sanft zum Umlenken zu bewegen.
34*	Nonsense	Ich finde groteske und phantastische Geschichten erheiternd.
35*	Irony	Ich habe Spass daran, wenn jemand das Gegenteil von dem sagt was er/sie meint und dabei erwartet, dass Eingeweihte es verstehen und Aussenstehende nicht.
36*	Cynicism	Im Allgemeinen sind die Menschen und die Welt schwach und es macht mir nichts aus, allgemein anerkannte Werte durch zynische Bemerkungen zu entwerten.
37*	Fun	Ich bin gerne närrisch.
38	Irony	Ich kann jemanden sanft kritisieren/tadeln indem ich ihn/sie vermeintlich lobe.
39	Cynicism	Ich bin skeptisch, dass die Menschen auch immer die guten Beweggründe für ihr Handeln haben, die sie so vorgeben. Deswegen lache ich darüber.
40*	Wit	Ich überrasche andere durch witzige Bemerkungen und treffende Urteile, die mir zu einem aktuellen Thema spontan einfallen.
41*	Sarcasm	Ich bin oft bösartig-kritisch, wenn ich das Korrupte, Verdorbene, Lasterhafte, bzw. Schlechte anprangere.
42*	Humor	Humor ist geeignet, Verständnis und auch Mitgefühl für das Unvollkommene und Menschliche zu wecken.
43*	Satire	Ich mache das moralisch Schlechte gerne lächerlich, um eine kritische Einstellung bei anderen Menschen zu erzeugen bzw. diese zu erhöhen.
44*	Nonsense	Absurditäten amüsieren mich.
45	Irony	Meine vertrauten Freunde verstehen mich, auch wenn ich mich verstelle und ironische Äußerungen mache.
46*	Cynicism	Menschen glauben zu naiv und blind an Werte und Moral, was meinem Spott und Hohn Nahrung gibt.
47*	Fun	Ich necke meine Freunde/Freundinnen gerne auf lustige Weise.
48	Irony	Meine Ironie enthält oft Bewertungen und ich gehe davon aus, dass der ironisch Kritisierte einsichtig wird und ich so auf eine offene direkte Kritik verzichten kann.

49*	Cynicism	Ich habe die Tendenz, nicht an die Aufrichtigkeit mancher Absichten und Werte zu glauben und stelle diese oft durch zynische Bemerkungen bloss.
50	Wit	Meine Zuhörer schätzen, dass ich mit pointierten Aussagen einen Sachverhalt schlagartig erhellen (wie durch ein Blitzlicht) und auf den Punkt bringen kann.
51*	Sarcasm	Ich verfüge über einen bitteren, beissenden Spott, den ich sowohl direkt als auch indirekt (z.B. mittels Ironie) ausdrücke.
52*	Humor	Auch bei unangenehmen Vorkommnissen kann ich Abstand zu Dingen halten, und etwas Erheiterndes bzw. Komisches daran entdecken.
53*	Satire	Wenn die Verhältnisse nicht so sind wie sie eigentlich sein sollten, nehme ich diese moralischen Verfehlungen oder die gesellschaftlichen Missstände aufs Korn, in der Hoffnung, sie nachhaltig zu verbessern.
54*	Nonsense	Ich mag Humor, der sich über die Grenzen der Logik hinwegsetzt.
55*	Irony	Meine Ironie bewirkt, dass sichtbar wird, wer schlau genug ist und etwas durchschaut und wer nicht.
56	Sarcasm	Ich liebe geschliffenen und beissenden Spott, auch wenn ich dabei gesellschaftliche Konventionen missachte bzw. die Gefühle anderer verletze.
57*	Fun	Ich mache gerne Scherze und bin albern.
58*	Irony	Wer meine Ironie versteht, ist mit mir überlegen gegenüber denen, die es nicht raffen (kapieren).
59*	Cynicism	Einige der moralischen Normen verachte ich und sehe sie zynisch, obwohl es mir nicht generell an einem Sinn für moralische Werte fehlt.
60*	Wit	Mein Witz und wacher Geist helfen mir schlagfertig zu sein.
61*	Sarcasm	Ich zeige gelegentlich bitteren Hohn.
62	Nonsense	Ich konstruiere gerne lustige Geschichten voller Ungereimtheiten und unauflösbarer Widersprüche (Paradoxien).
63*	Cynicism	Ich habe eine zynische Haltung zu einigen gängigen Normen und Moralvorstellungen; ich glaube nicht an sie und finde sie zumeist lächerlich.
64*	Irony	Wenn ich etwas Ironisches sage, dann gibt es immer jemanden in meiner Gruppe der das versteht und andere, die das nicht tun.

65	Fun	Meine Spässe können gelegentlich etwas derb sein, aber meine Freunde/Freundinnen schätzen das.
66*	Irony	Unsere Ironie verwirrt diejenigen, welche sie nicht verstehen, da ich und meine Vertrauten in Schwebe halten, was wir wirklich meinen.
67	Wit	Ich kann einen scharfen Blick auf Alltagssituationen und die Beteiligten werfen und darin etwas unfreiwillig Komisches entdecken und witzig darstellen.
68	Sarcasm	Gelegentlich lache ich Leute aus, um sie zu demütigen, da sie es wirklich verdienen.
69	Nonsense	Ich mag Schüttelreime (z.B. „Er schüttelte die Klapperschlang', bis ihre Klapper schlapper klang“) und Unsinnsgedichte.
70*	Cynicism	Ich neige dazu, vor gewissen Moral- und Wertvorstellungen keine Ehrfurcht aufzubringen, sondern nur Hohn und Häme.
71	Fun	Ich mache gerne Witze zur Belustigung meiner Freunde.
72*	Wit	Ich kann Beziehungen zwischen unzusammenhängenden Ideen oder Gedanken herstellen und damit kurz und pointiert eine komische Wirkung erzeugen.
73*	Sarcasm	Mein Lachen ist gelegentlich hämisch und drückt Schadenfreude aus.

Notes. * denotes items retained for the final version of the Comic Style Markers.

Table S3

Means and Standard Deviations of the Comic Style Markers (CSM) employed in Studies 1 and 2

	Study 1								Study 2					
	Sample 3 pretest (*N* = 148)		Sample 3 posttest (*N* = 148)		Sample 4 (*N* = 210)		Sample 4 other-reports (*N* = 210)		Sample 1 (*N* = 999)		Sample 2 (*N* = 252)		Sample 3 (*N* = 214)	
CSM	*M*	*SD*	*M*	*SD*	*M*	*SD*	*M*	*SD*	*M*	*SD*	*M*	*SD*	*M*	*SD*
Fun	4.47	1.13	4.50	1.14	4.44	1.07	4.54	0.93	4.37	1.16	4.02	1.17	4.69	0.84
Humor	5.14	0.83	5.01	0.88	5.01	0.81	4.78	0.70	5.01	0.83	4.77	0.87	5.13	0.74
Nonsense	4.93	1.19	4.83	1.37	4.75	1.12	4.62	0.88	4.92	1.10	4.53	1.18	4.79	1.14
Wit	4.73	1.04	4.80	1.07	4.70	1.03	5.04	0.80	4.83	1.05	4.43	1.12	4.78	0.88
Irony	4.45	1.11	4.33	1.25	4.42	1.04	4.40	0.88	4.46	1.16	4.23	1.17	4.35	0.98
Satire	4.04	1.04	3.85	1.19	3.96	1.00	4.03	0.72	4.22	1.02	3.91	1.07	3.99	0.97
Sarcasm	3.61	1.27	3.45	1.39	3.68	1.25	3.38	1.11	3.66	1.33	3.55	1.29	3.63	1.13
Cynicism	3.92	1.40	3.73	1.51	3.83	1.29	3.45	1.01	3.84	1.33	3.65	1.37	3.67	1.09

Table S4

Means and Standard Deviations of the Criteria and Zero-Order Correlations of the Comic Style Markers with the Criteria from Study 2

Variables	*M*	*SD*	Fun	Humor	Non.	Wit	Irony	Satire	Sarc.	Cyn.
MRS-25 (*N* = 999)										
Extraversion	4.15	1.00	.40***	.30***	.14***	.37***	.06*	.16***	-.04	-.09**
Agreeableness	4.37	0.77	.04	.25***	.05	.00	-.17***	-.11***	-.41***	-.32***
Conscientiousness	4.12	0.91	-.14***	-.09**	-.15***	-.08*	-.04	-.11***	-.09**	-.13***
Emotional stability	3.82	0.96	.19***	.35***	.13***	.30***	.03	.08**	-.09**	-.02
Culture	4.43	0.79	.24***	.34***	.28***	.40***	.08*	.21***	-.03	.04
Strength factors (*N* = 252)										
Emotional	0.00	1.00	.41***	.41***	.26***	.49***	.25***	.25***	.19***	.07
Interpersonal	0.00	1.00	.11	.11	-.03	-.01	-.19**	-.09	-.27***	-.28***
Restraint	0.01	1.00	-.30***	-.13*	-.22***	-.16**	-.13*	-.18**	-.21***	-.18**
Intellectual	0.01	1.00	.00	.01	-.02	-.12	-.24***	-.15*	-.39***	-.33***
Theological	0.00	1.00	.11	.22***	.28***	.45***	.24***	.27***	.13*	.23***
Measured intelligence (*N* = 199)										
Verbal	111.20	7.34	.06	.09	.07	.19**	.10	-.02	.08	.00
Numerical	108.06	7.94	.03	.14*	.02	.04	.10	.15*	.04	.11
Spatial	101.55	9.27	-.07	-.02	.05	-.04	.08	.03	-.05	.09
Total	109.31	6.87	-.02	.08	.04	.09	.12	.10	.03	.09
Self-rated intelligence (*N* = 214)										
Verbal	40.52	11.34	.09	.29***	.09	.48***	.03	.14*	.05	-.01
Numerical	31.96	13.53	.01	.10	.08	.05	.14*	.08	.07	.14*
Spatial	32.61	15.45	-.02	.07	.11	.09	.16*	.11	-.07	.13
Total	34.96	8.99	.03	.21**	.13*	.28***	.17*	.16*	.02	.14*

Note. Non. = nonsense, Sarc. = sarcasm, Cyn. = cynicism.

$^{*}p < .05$. $^{**}p < .01$. $^{***}p < .001$.

Table S5

Partial Correlations and Multiple Regressions between Character Strengths (VIA-Inventory of Strengths Scales) and the Comic Style Markers (Controlled for Age and Gender)

VIA-IS scales	Fun	Humor	Non.	Wit	Irony	Satire	Sarc.	Cyn.	*R*	Adj. R^2
	Virtue I *wisdom and knowledge*: Cognitive strengths that entail the acquisition and use of knowledge.									
Creativity	.26***	.33***	.33***	.54***	.20**	.31***	.09	.17**	.59	.32
Curiosity	.26***	.34***	.25***	.40***	.10	.17**	-.05	-.07	.49	.21
Open-mindedness	.05	.20**	.08	.34***	.19**	.17**	.08	.11	.41	.14
Love of learning	.12	.23***	.23***	.38***	.18**	.14*	.06	.09	.41	.14
	Virtue II *courage*: Emotional strengths that involve the exercise of will to accomplish goals in the face of opposition, external or internal									
Perspective	.17**	.24***	.17**	.41***	.25***	.18**	.04	.07	.47	.19
Bravery	.25***	.34***	.30***	.44***	.15*	.24***	.14*	.09	.47	.20
Perseverance	.13*	.14*	.10	.24***	.08	.05	.03	-.11	.32	.08
Honesty	.08	.18**	.02	.14*	.06	.01	-.07	-.11	.30	.06
Zest	.39***	.41***	.25***	.40***	.11	.21***	-.02	-.07	.55	.28
	Virtue III *humanity*: Interpersonal strengths that involve "tending and befriending" others									
Love	.33***	.21***	.15*	.26***	-.02	.02	-.16*	-.23***	.53	.25
Kindness	.30***	.24***	.17**	.21**	.00	.05	-.12	-.19**	.47	.19
Social intelligence	.33***	.37***	.22***	.38***	.13*	.17**	-.02	-.06	.50	.23
	Virtue IV *justice*: Civic strengths that underlie healthy community life									
Teamwork	.22***	.23**	.06	.15*	-.09	-.06	-.21**	-.28***	.50	.22
Fairness	-.03	.03	-.08	-.08	-.29***	-.10	-.31***	-.28***	.41	.14
Leadership	.25***	.25***	.06	.32***	.02	.12	-.12	-.15*	.50	.23
	Virtue V *temperance*: Strengths that protect against excess									
Forgiveness	.07	.15*	.03	.05	-.18**	-.04	-.34***	-.26***	.50	.22
Humility	-.06	.00	-.05	-.19**	-.07	-.12	-.20**	-.15*	.34	.09
Prudence	-.14*	-.04	-.11	-.03	-.01	-.03	-.18**	-.14*	.32	.07
Self-regulation	.07	.12	.01	.20**	.05	.01	-.08	-.11	.31	.07
	Virtue VI *transcendence*: Strengths that forge connections to the larger universe and provide meaning									
Beauty	.24***	.17**	.25***	.31***	.09	.16*	-.10	-.01	.41	.14
Gratitude	.21***	.15*	.03	.13*	.02	.03	-.19**	-.19**	.44	.17
Hope	.30***	.36***	.17**	.32***	.05	.14*	-.06	-.11	.49	.21
Humor	.63***	.58***	.38***	.61***	.33***	.39***	.15*	.13*	.76	.56
Religiousness	.15*	.13*	.08	.11	-.05	.01	-.23***	-.17*	.41	.14
R	.70	.66	.57	.80	.66	.57	.63	.64		
Adj. R^2	.43	.38	.25	.61	.38	.26	.33	.34		

Notes. $N = 252$. Beauty = appreciation of beauty and excellence, Non. = nonsense, Sarc. = sarcasm, Cyn. = cynicism. Virtue descriptions adapted from Peterson and Seligman (2004).

$^{*}p < .05$. $^{**}p < .01$. $^{***}p < .001$.

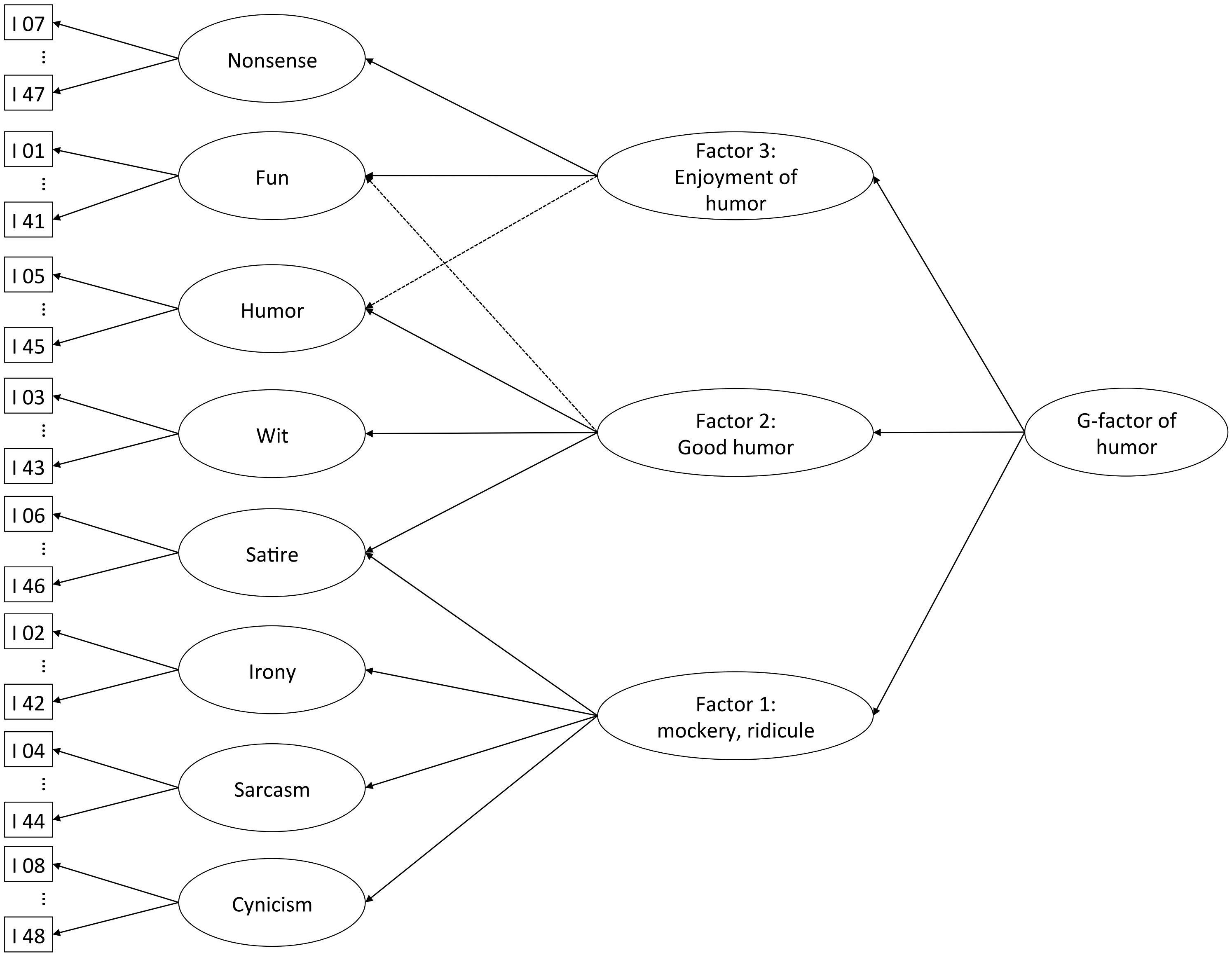

Figure S1. Schematic representation of the factor-analytic results of Study 1.
